# Common breast cancer susceptibility alleles are associated with tumour subtypes in *BRCA1 *and *BRCA2 *mutation carriers: results from the Consortium of Investigators of Modifiers of *BRCA1/2*

**DOI:** 10.1186/bcr3052

**Published:** 2011-11-02

**Authors:** Anna Marie Mulligan, Fergus J Couch, Daniel Barrowdale, Susan M Domchek, Diana Eccles, Heli Nevanlinna, Susan J Ramus, Mark Robson, Mark Sherman, Amanda B Spurdle, Barbara Wappenschmidt, Andrew Lee, Lesley McGuffog, Sue Healey, Olga M Sinilnikova, Ramunas Janavicius, Thomas vO Hansen, Finn C Nielsen, Bent Ejlertsen, Ana Osorio, Iván Muñoz-Repeto, Mercedes Durán, Javier Godino, Maroulio Pertesi, Javier Benítez, Paolo Peterlongo, Siranoush Manoukian, Bernard Peissel, Daniela Zaffaroni, Elisa Cattaneo, Bernardo Bonanni, Alessandra Viel, Barbara Pasini, Laura Papi, Laura Ottini, Antonella Savarese, Loris Bernard, Paolo Radice, Ute Hamann, Martijn Verheus, Hanne EJ Meijers-Heijboer, Juul Wijnen, Encarna B Gómez García, Marcel R Nelen, C Marleen Kets, Caroline Seynaeve, Madeleine MA Tilanus-Linthorst, Rob B van der Luijt, Theo van Os, Matti Rookus, Debra Frost, J Louise Jones, D Gareth Evans, Fiona Lalloo, Ros Eeles, Louise Izatt, Julian Adlard, Rosemarie Davidson, Jackie Cook, Alan Donaldson, Huw Dorkins, Helen Gregory, Jacqueline Eason, Catherine Houghton, Julian Barwell, Lucy E Side, Emma McCann, Alex Murray, Susan Peock, Andrew K Godwin, Rita K Schmutzler, Kerstin Rhiem, Christoph Engel, Alfons Meindl, Ina Ruehl, Norbert Arnold, Dieter Niederacher, Christian Sutter, Helmut Deissler, Dorothea Gadzicki, Karin Kast, Sabine Preisler-Adams, Raymonda Varon-Mateeva, Ines Schoenbuchner, Britta Fiebig, Wolfram Heinritz, Dieter Schäfer, Heidrun Gevensleben, Virginie Caux-Moncoutier, Marion Fassy-Colcombet, François Cornelis, Sylvie Mazoyer, Mélanie Léoné, Nadia Boutry-Kryza, Agnès Hardouin, Pascaline Berthet, Danièle Muller, Jean-Pierre Fricker, Isabelle Mortemousque, Pascal Pujol, Isabelle Coupier, Marine Lebrun, Caroline Kientz, Michel Longy, Nicolas Sevenet, Dominique Stoppa-Lyonnet, Claudine Isaacs, Trinidad Caldes, Miguel de la Hoya, Tuomas Heikkinen, Kristiina Aittomäki, Ignacio Blanco, Conxi Lazaro, Rosa B Barkardottir, Penny Soucy, Martine Dumont, Jacques Simard, Marco Montagna, Silvia Tognazzo, Emma D'Andrea, Stephen Fox, Max Yan, Tim Rebbeck, Olufunmilayo I Olopade, Jeffrey N Weitzel, Henry T Lynch, Patricia A Ganz, Gail E Tomlinson, Xianshu Wang, Zachary Fredericksen, Vernon S Pankratz, Noralane M Lindor, Csilla Szabo, Kenneth Offit, Rita Sakr, Mia Gaudet, Jasmine Bhatia, Noah Kauff, Christian F Singer, Muy-Kheng Tea, Daphne Gschwantler-Kaulich, Anneliese Fink-Retter, Phuong L Mai, Mark H Greene, Evgeny Imyanitov, Frances P O'Malley, Hilmi Ozcelik, Gordon Glendon, Amanda E Toland, Anne-Marie Gerdes, Mads Thomassen, Torben A Kruse, Uffe Birk Jensen, Anne-Bine Skytte, Maria A Caligo, Maria Soller, Karin Henriksson, von Anna Wachenfeldt, Brita Arver, Marie Stenmark-Askmalm, Per Karlsson, Yuan Chun Ding, Susan L Neuhausen, Mary Beattie, Paul DP Pharoah, Kirsten B Moysich, Katherine L Nathanson, Beth Y Karlan, Jenny Gross, Esther M John, Mary B Daly, Saundra M Buys, Melissa C Southey, John L Hopper, Mary Beth Terry, Wendy Chung, Alexander F Miron, David Goldgar, Georgia Chenevix-Trench, Douglas F Easton, Irene L Andrulis, Antonis C Antoniou

**Affiliations:** 1 Department of Laboratory Medicine and Pathobiology, University of Toronto, Toronto, ON M5S 1A8, Canada; Department of Laboratory Medicine, and the Keenan Research Centre of the Li Ka Shing Knowledge Institute, St Michael's Hospital, 30 Bond Street, Toronto, ON M5B 1W8, Canada; 2 Department of Laboratory Medicine and Pathology, Mayo Clinic, 200 First Street SW, Rochester, MN 55905, USA; 3 Centre for Cancer Genetic Epidemiology, Department of Public Health and Primary Care, University of Cambridge, 2 Worts Causeway, Cambridge, CB1 8RN, UK; 4 Department of Medicine, Abramson Cancer Center, Perelman School of Medicine at the University of Pennsylvania, 3400 Civic Center Boulevard, Philadelphia, PA 19104, USA; 5 Faculty of Medicine, University of Southampton, University Hospital Southampton NHS Foundation Trust, Tremona Road, Southampton, SO16 6YD, UK; 6 Department of Obstetrics and Gynecology, University of Helsinki and Helsinki University Central Hospital, Haartmaninkatu 8, 00290 Helsinki, Finland; 7 Department of Preventive Medicine, Keck School of Medicine, University of Southern California, 2001 N Soto Street, Los Angeles, CA 90089-9237, USA; 8 Department of Medicine, Memorial Sloan-Kettering Cancer Center and Weill Cornell Medical College, 1275 York Ave, New York, NY 10065, USA; 9 National Cancer Institute, Division of Cancer Epidemiology and Genetics, Hormonal and Reproductive Epidemiology Branch, 6120 Executive Blvd., Rockville, MD 20852, USA; 10 Queensland Institute of Medical Research, 300 Herston Rd, Herston, Brisbane, QLD 4006, Australia; 11 Centre of Familial Breast and Ovarian Cancer, Department of Gynaecology and Obstetrics and Centre for Integrated Oncology (CIO), University Hospital of Cologne, Kerpener Str. 62, Cologne, 50931, Germany; 12 Unité Mixte de Génétique Constitutionnelle des Cancers Fréquents, Centre Hospitalier Universitaire de Lyon/Centre Léon Bérard, 28 rue Laënnec, Lyon 69373, France; 13 INSERM U1052, CNRS UMR5286, Université Lyon 1, Cancer Research Center of Lyon, 28 rue Laënnec, Lyon 69373, France; 14 Department of Molecular and Regenerative Medicine, Hematology, Oncology and Transfusion Medicine Center, Vilnius University Hospital Santariskiu Clinics, Santariskiu st 2, LT-08661 Vilnius and State Research Institute Innovative Medicine Center, Zygimantu st. 9, LT-01102 Vilnius, Lithuania; 15 Center for Genomic Medicine, Rigshospitalet, Copenhagen University Hospital, Blegdamsvej 9, DK-2100 Copenhagen, Denmark; 16 Department of Oncology, Rigshospitalet Bldg. 4262, Copenhagen University Hospital, Blegdamsvej 9, DK-2100 Copenhagen, Denmark; 17 Human Genetics Group, Human Cancer Genetics Programme, Spanish National Cancer Research Centre, C/Melchor Fernández Almagro 3, Madrid, 28029, Spain and the Spanish Network on Rare Diseases (CIBERER); 18 Institute of Biology and Molecular Genetics, Universidad de Valladolid (IBGM-UVA), C/Sanz y Forés, N° 3, Valladolid, 47003, Spain; 19 Instituto de investigación sanitaria de Aragón (IIS), Hospital clinico Universitario "Lozano Blesa", San Juan Bosco 15, Zaragoza, 50009, Spain; 20 Molecular Diagnostics Laboratory, IRRP, National Center for Scientific Research Demokritos, Patriarchou Gregoriou E' & Neapoleos Str, Ag. Paraskevi 15310, Athens, Greece; 21 Human Genetics Group and Genotyping Unit, Human Cancer Genetics Programme, Spanish National Cancer Research Centre, C/Melchor Fernández Almagro 3, Madrid, 28029, Spain and the Spanish Network on Rare Diseases (CIBERER); 22 Unit of Molecular Bases of Genetic Risk and Genetic Testing, Department of Preventive and Predictive Medicine, Fondazione IRCCS Istituto Nazionale Tumouri (INT), via Giacomo Venezian 1, 20133 Milan, Italy; IFOM, Fondazione Istituto FIRC di Oncologia Molecolare, via Adamello 16, 20139 Milan, Italy; 23 Unit of Medical Genetics, Department of Preventive and Predictive Medicine, Fondazione IRCCS Istituto Nazionale Tumouri (INT), via Giacomo Venezian 1, Milan, 20133, Italy; 24 Division of Cancer Prevention and Genetics, Istituto Europeo di Oncologia, via Ripamonti 435, Milan, 20141, Italy; 25 Unit of Experimental Oncology 1, Centro di Riferimento Oncologico, IRCCS, Aviano (PN), Italy; 26 Department of Genetics, Biology and BiochemIstry, University of Turin, Turin, Italy; 27 Medical Genetics Unit, Department of Clinical Physiopathology, University of Florence, Firenze, Italy; 28 Department of Molecular Medicine, "Sapienza" University of Rome, Rome, Italy; 29 Division of Medical Oncology, Regina Elena Cancer Institute, Rome, Italy; 30 Department of Experimental Oncology, Istituto Europeo di Oncologia. Milan, Italy; Consortium for Genomics Technology (Cogentech), Milan, Italy; 31 Molecular Genetics of Breast Cancer, Deutsches Krebsforschungszentrum (DKFZ), Heidelberg, Germany; 32 Department of Epidemiology, Netherlands Cancer Institute, Amsterdam, The Netherlands; 33 Department of Clinical Genetics, VU Medical Center, Amsterdam, The Netherlands; 34 Department of Human Genetics and Department of Clinical Genetics, Leiden University Medical Center, Leiden, The Netherlands; 35 Department of Clinical Genetics and GROW, School for Oncology and Developmental Biology, MUMC, Maastricht, The Netherlands; 36 Department of Human Genetics 849, Radboud University Nijmegen Medical Centre, P.O. BOX 9101, 6500 HB Nijmegen, The Netherlands; 37 Department of Human Genetics 836, Radboud University Nijmegen Medical Centre, P.O. BOX 9101, 6500 HB Nijmegen, The Netherlands; 38 Department of Medical Oncology, Family Cancer Clinic, Erasmus University Medical Center, Rotterdam, The Netherlands; 39 Department of Surgical Oncology, Family Cancer Clinic, Erasmus University Medical Center, Rotterdam, The Netherlands; 40 Department of Medical Genetics, University Medical Center Utrecht, The Netherlands; 41 Department of Clinical Genetics, Academic Medical Center, Amsterdam, The Netherlands; 42 Barts Cancer Institute, Queen Mary University of London, Centre for Tumour Biology, Charterhouse Square, London, UK; 43 Genetic Medicine, Manchester Academic Health Sciences Centre, Central Manchester University Hospitals NHS Foundation Trust, Manchester, UK; 44 Oncogenetics Team, The Institute of Cancer Research and Royal Marsden NHS Foundation Trust, UK; 45 Clinical Genetics, Guy's and St. Thomas' NHS Foundation Trust, London, UK; 46 Yorkshire Regional Genetics Service, Leeds, UK; 47 Ferguson-Smith Centre for Clinical Genetics, Yorkhill Hospitals, Glasgow, UK; 48 Sheffield Clinical Genetics Service, Sheffield Children's Hospital, Sheffield, UK; 49 Clinical Genetics Department, St Michael's Hospital, Bristol, UK; 50 North West Thames Regional Genetics Service, Kennedy-Galton Centre, Harrow, UK; 51 North of Scotland Regional Genetics Service, NHS Grampian and University of Aberdeen, Foresterhill, Aberdeen, UK; 52 Nottingham Clinical Genetics Service, Nottingham University Hospitals NHS Trust, UK; 53 Cheshire and Merseyside Clinical Genetics Service, Liverpool Women's NHS Foundation Trust, Liverpool, UK; 54 Leicestershire Clinical Genetics Service, University Hospitals of Leicester NHS Trust, UK; 55 North East Thames Regional Genetics Service, Great Ormond Street Hospital for Children NHS Trust, London, UK; 56 All Wales Medical Genetics Service, Glan Clwyd Hospital, Rhyl, UK; 57 All Wales Medical Genetics Services, Singleton Hospital, Swansea, UK; 58 Department of Pathology and Laboratory Medicine, University of Kansas Medical Center, Kansas City, KS, USA; 59 Institute for Medical Informatics, Statistics and Epidemiology, University of Leipzig, Leipzig, Germany; 60 Department of Gynaecology and Obstetrics, Division of Tumour Genetics, Klinikum rechts der Isar, Technical University, Munich, Germany; 61 Department of Gynaecology and Obstetrics, Ludwig-Maximillians University, Munich, Germany; 62 Department of Gynaecology and Obstetrics, University Hospital of Schleswig-Holstein (UKSH), Campus Kiel, Christian-Albrechts University, Kiel, Germany; 63 Department of Gynaecology and Obstetrics, University Hospital Düsseldorf, Heinrich-Heine University, Düsseldorf, Germany; 64 Institute of Human Genetics, Department of Human Genetics, Heidelberg University Hospital, Heidelberg, Germany; 65 Department of Gynaecology and Obstetrics, University Hospital, Ulm, Germany; 66 Institute of Cell and Molecular Pathology, Hannover Medical School, Hannover, Germany; 67 Department of Gynaecology and Obstetrics, University Hospital Carl Gustav Carus, Technical University, Dresden, Germany; 68 Institute of Human Genetics, University of Münster, Münster, Germany; 69 Institute of Human Genetics, Campus Virchov Klinikum, Charite Berlin, Germany; 70 Centre of Familial Breast and Ovarian Cancer, Department of Medical Genetics, Institute of Human Genetics, University Würzburg, Germany; 71 Institute of Human Genetics, University Regensburg, Germany; 72 Institute of Human Genetics, University Leipzig, Germany; 73 Institute of Human Genetics, University Hospital, Frankfurt a.M., Germany; 74 Breakthrough Breast Cancer Research Centre, Institute of Cancer Research, UK; 75 Service de Génétique Oncologique, Institut Curie, Paris, France; 76 Genetic Unit, Avicenne Hospital, Assitance Publique-Hôpitaux de Paris, Paris, France; Sud-Francilien Hospital, Evry-Corbeil, France; University Hospital, Clermont-Ferrand, France; 77 Centre François Baclesse, Caen, France; 78 Unité d'Oncogénétique, CLCC Paul Strauss, Strasbourg, France; 79 Service de Génétique, Centre Hospitalier Universitaire Bretonneau, Tours, France; 80 Unité d'Oncogénétique, CHU Arnaud de Villeneuve, Montpellier, France; 81 Service de Génétique Clinique Chromosomique et Moléculaire, Centre Hospitalier Universitaire de St Etienne, St Etienne, France; 82 Cancer Genetics Unit, INSERM U916, Institut Bergonié, Université de Bordeaux, Bordeaux, France; 83 Unité INSERM U830, Institut Curie, Paris, France; Université Paris Descartes, Faculté de Médecine, Paris, France; 84 Lombardi Comprehensive Cancer Center, Georgetown University, Washington DC, USA; 85 Molecular Oncology Laboratory, Hospital Clinico San Carlos, Madrid, Spain; 86 Department of Clinical Genetics, Helsinki University Central Hospital, Meilahdentie 2, 00290 Helsinki, Finland; 87 Hereditary Cancer Program, Institut Català d'Oncologia, Hospital Duran i Reynals - Bellvitge Biomedical Research Institute (IDIBELL), L'Hospitalet de Llobregat, Barcelona, Spain; 88 Department of Pathology, Landspitali University Hospital, Reykjavik, Iceland, Faculty of Medicine, University of Iceland, Reykjavik, Iceland; 89 Cancer Genomics Laboratory, Centre Hospitalier Universitaire de Québec, 2705 Laurier Boulevard, T3-57, Quebec City, QC, Canada; 90 Cancer Genomics Laboratory, Centre Hospitalier Universitaire de Québec, 2705 Laurier Boulevard, T3-57, Quebec City, QC, Canada; Canada Research Chair in Oncogenetics, Department of Molecular Medicine, Faculty of Medicine, Laval University, QC, Canada; 91 Immunology and Molecular Oncology Unit, Istituto Oncologico Veneto IOV - IRCCS, Via Gattamelata 64, 35128 Padua, Italy; 92 Department of Oncology and Surgical Sciences, University of Padua and Istituto Oncologico Veneto IOV - IRCCS, Via Gattamelata 64, 35128 Padua, Italy; 93 Peter MacCallum Cancer Centre, Melbourne, VIC 3052, Australia; 94 Department of Anatomical Pathology, Prince of Wales Hospital, Randwick, NSW 2031, Australia; 95 Abramson Cancer Center and University of Pennsylvania Perelman School of Medicine, Philadelphia, PA, USA; 96 University of Chicago, Chicago, IL, USA; 97 City of Hope Comprehensive Cancer Center and Department of Population Sciences, Beckman Research Institute, City of Hope, Duarte, CA, USA; 98 Departments of Medicine, and Preventive Medicine and Public Health, Creighton University, Omaha, NE, USA; 99 Jonsson Comprehensive Cancer Center at the University of California, Los Angeles, CA, USA; 100 Department of Internal Medicine and Harold C. Simmons Comprehensive Cancer Center, University of Texas, Southwestern Medical Center, Dallas, TX; USA; Department of Pediatrics, University of Texas Health Science Center at San Antonio, San Antonio, TX, USA; 101 Department of Health Sciences Research, Mayo Clinic, Rochester, MN, USA; 102 Department of Medical Genetics, Mayo Clinic, Rochester, MN, USA; 103 University of Delaware, Newark, DE, USA; 104 Epidemiology Research Program, American Cancer Society, Atlanta, GA, USA; 105 Department of Obstetrics/Gynaecology and Comprehensive Cancer Center, Medical University of Vienna, Vienna, Austria; 106 Clinical Genetics Branch, Division of Cancer Epidemiology and Genetics, US National Cancer Institute, Rockville, MD, USA; 107 Laboratory of Molecular Oncology, N.N. Petrov Institute of Oncology, St.-Petersburg, Russia; 108 Department of Laboratory Medicine and Pathobiology, University of Toronto, Toronto, ON, Canada; Department of Laboratory Medicine, and the Keenan Research Centre of the Li Ka Shing Knowledge Institute, St Michael's Hospital, Toronto, ON, Canada; 109 Samuel Lunenfeld Research Institute, Mount Sinai Hospital, Toronto; Department of Laboratory Medicine and Pathobiology, University of Toronto, ON, Canada; 110 Cancer Care Ontario, ON, Canada; 111 Departments of Molecular Virology, Immunology and Medical Genetics and Internal Medicine, The Ohio State University Comprehensive Cancer Center, Columbus, OH, USA; 112 Clinical Genetics, Rigshospital, Copenhagen University, Copenhagen, Denmark; 113 Clinical Genetics, Odense University Hospital, Odense, Denmark; 114 Department of Clinical Genetics, Aarhus University Hospital, Aarhus, Denmark; 115 Clinical Genetics, Vejle Hospital, Denmark; 116 Section of Genetic Oncology, Dept. of Laboratory Medicine, University and University Hospital of Pisa, Pisa, Italy; 117 Department of Clinical Genetics, Lund University Hospital, Lund, Sweden; 118 Oncological Centre, Lund University Hospital, Lund, Sweden; 119 Department of Oncology, Karolinska University Hospital, Stockholm, Sweden; 120 Division of Clinical Genetics, Department of Clinical and Experimental Medicine, Linköping University, Linköping, Sweden; 121 Department of Oncology, Sahlgrenska University Hospital, Gothenburg, Sweden; 122 Department of Population Sciences, the Beckman Research Institute of the City of Hope, Duarte, CA, USA; 123 UCSF Cancer Risk Program and Departments of Medicine and Epidemiology and Biostatistics, University of California San Francisco, San Francisco, CA, USA; 124 Department of Oncology, University of Cambridge, Cambridge, UK; 125 Department of Cancer Prevention and Control, Roswell Park Cancer Institute, Buffalo, NY, USA; 126 Women's Cancer Program at the Samuel Oschin Comprehensive Cancer Institute, Cedars-Sinai Medical Center, Los Angeles, CA, USA; 127 Department of Epidemiology, Cancer Prevention Institute of California, Fremont, CA, USA; 128 Fox Chase Cancer Center, Philadelphia, PA, USA; 129 Department of Oncological Sciences, Huntsman Cancer Institute, University of Utah, Salt Lake City, UT, USA; 130 Genetic Epidemiology Laboratory, Department of Pathology, University of Melbourne, Melbourne, VIC, Australia; 131 Centre for Molecular, Environmental, Genetic and Analytic Epidemiology, University of Melbourne, Melbourne, VIC, Australia; 132 Department of Epidemiology, Columbia University, New York, NY, USA; 133 Department of Cancer Biology, Dana-Farber Cancer Institute, Boston, MA, USA; 134 Department of Dermatology, University of Utah School of Medicine, Salt Lake City, UT, USA; 135 Samuel Lunenfeld Research Institute, Mount Sinai Hospital, 600 University Avenue, Toronto, ON, M5G 1X5, Canada; Cancer Care Ontario, Departments of Molecular Genetics and Laboratory Medicine and Pathobiology, University of Toronto, ON, Canada; 136 Breast Cancer Family Registry; 137 Cancer Genetics Network "Groupe Génétique et Cancer", Fédération Nationale des Centres de Lutte Contre le Cancer; 138 Netherlands Cancer Institute, Amsterdam, The Netherlands; 139 Ontario Cancer Genetics Network, Cancer Care Ontario, 620 University Avenue, Toronto, ON M5G 2L7, Canada; 140 Karolinska Institute, Stockholm, Sweden

## Abstract

**Introduction:**

Previous studies have demonstrated that common breast cancer susceptibility alleles are differentially associated with breast cancer risk for *BRCA1 *and/or *BRCA2 *mutation carriers. It is currently unknown how these alleles are associated with different breast cancer subtypes in *BRCA1 *and *BRCA2 *mutation carriers defined by estrogen (ER) or progesterone receptor (PR) status of the tumour.

**Methods:**

We used genotype data on up to 11,421 *BRCA1 *and 7,080 *BRCA2 *carriers, of whom 4,310 had been affected with breast cancer and had information on either ER or PR status of the tumour, to assess the associations of 12 loci with breast cancer tumour characteristics. Associations were evaluated using a retrospective cohort approach.

**Results:**

The results suggested stronger associations with ER-positive breast cancer than ER-negative for 11 loci in both *BRCA1 *and *BRCA2 *carriers. Among *BRCA1 *carriers, single nucleotide polymorphism (SNP) rs2981582 (*FGFR2) *exhibited the biggest difference based on ER status (per-allele hazard ratio (HR) for ER-positive = 1.35, 95% CI: 1.17 to 1.56 vs HR = 0.91, 95% CI: 0.85 to 0.98 for ER-negative, *P*-heterogeneity = 6.5 × 10^-6^). In contrast, SNP rs2046210 at 6q25.1 near *ESR1 *was primarily associated with ER-negative breast cancer risk for both *BRCA1 *and *BRCA2 *carriers. In *BRCA2 *carriers, SNPs in *FGFR2, TOX3, LSP1, SLC4A7/NEK10*, 5p12, 2q35, and 1p11.*2 *were significantly associated with ER-positive but not ER-negative disease. Similar results were observed when differentiating breast cancer cases by PR status.

**Conclusions:**

The associations of the 12 SNPs with risk for *BRCA1 *and *BRCA2 *carriers differ by ER-positive or ER-negative breast cancer status. The apparent differences in SNP associations between *BRCA1 *and *BRCA2 *carriers, and non-carriers, may be explicable by differences in the prevalence of tumour subtypes. As more risk modifying variants are identified, incorporating these associations into breast cancer subtype-specific risk models may improve clinical management for mutation carriers.

## Introduction

Germline mutations in *BRCA1 *and *BRCA2 *confer high risks of breast, ovarian and other cancers [1-3] and account for 15 to 20% of the excess familial risk of breast cancer among first degree relatives [4,5]. Breast cancer risks for *BRCA1 *and *BRCA2 *mutation carriers have been estimated to range between 40 and 87% by age 70 [6-12] with population-based estimates tending to be lower than estimates based on families with multiple affected individuals [6,8]. Moreover, breast cancer risks for mutation carriers were found to vary according to the age at diagnosis and the type of cancer of the index patient involved in the family ascertainment [6,7,11]. Such evidence suggests that genetic or other risk factors that cluster in families modify the cancer risks conferred by *BRCA*1 and *BRCA2 *mutations.

A substantial body of work indicates that tumours arising in patients with germline *BRCA1 *mutations are morphologically and genetically distinct from those arising in carriers of *BRCA2 *mutations and from tumours in patients lacking mutations. In gene expression studies, *BRCA1*-associated tumours are often classified as basal subtype tumours [13,14]. This is reflected in their higher grade, and morphologic features including lymphocytic infiltrate, pushing margins and syncytial growth. Being basal-like they express several markers that are normally expressed in the basal/myoepithelial cells of the breast, including stratified epithelial cytokeratins 5/6, 14 and 17. *BRCA1*-associated tumours are more likely to be estrogen receptor (ER), progesterone receptor (PR) and HER2 negative and to harbor mutations in the *TP53 *gene than age-matched sporadic breast cancers [15,16]. *BRCA2*-associated tumours are also predominantly high-grade invasive ductal carcinomas of no special type but they often demonstrate a luminal phenotype despite their high histologic grade [13,17]. Adjusting for grade, *BRCA2*-associated tumours are more often ER-positive and are less likely, compared with controls, to express the basal cytokeratin CK5 or to overexpress HER2/neu protein [17].

Establishing the estrogen receptor status of a breast cancer (positive or negative) reflects a major subdivision in breast cancer type (at least five major sub-types are recognized) and it is becoming clear that the risk factors associated with breast cancer, both genetic and epidemiological, differ according to sub-type. Genome-wide association studies (GWAS) in breast cancer have identified several common alleles (single nucleotide polymorphisms (SNPs)) associated with an increased risk of breast cancer in the general population [18-25]. Many of these SNPs are associated with risk for ER-positive breast cancer, fewer have so far been associated with ER-negative breast cancer risk [26,27].

Known risk breast cancer susceptibility alleles have been genotyped in a large series of female *BRCA1 *and *BRCA2 *mutation carriers assembled by the Consortium of Investigators of Modifiers of BRCA1/2 (CIMBA) to evaluate their associations with risk of breast cancer for mutation carriers. Of the 12 SNPs (rs2981582 in *FGFR2*, rs3803662 in *TOX3/TNRC9*, rs889312 in *MAP3K1*, rs13281615 at 8q24, rs381798 in *LSP1*, rs13387042 at 2q35, rs4973768 in *SLC4A7/NEK10*, rs10941679 at 5p12, rs6504950 in *STXBP4/COX11*, rs999737/rs10483813 in *RAD51L1*, rs2046210 at 6q25.1 and rs11249433 at 1p11.2) investigated so far, eight were associated with breast cancer risk for *BRCA2 *carriers (all but SNPs at 8q24, *RAD51L1*, 6q25.1 and *STXBP4/COX11*), whereas only three SNPs (6q25.1, *TOX3/TNRC9 *and 2q35) were associated with risk for *BRCA1 *mutation carriers [28-31]. Work from the Breast Cancer Association Consortium and subsequent studies have demonstrated differences in the associations between these susceptibility loci and tumour characteristics in the general population [27,32]. These results suggest that the observed differences in the associations between *BRCA1 *and *BRCA2 *mutation carriers may reflect differences in the distribution of tumour characteristics in mutation carriers. It is currently unclear whether these polymorphisms are associated with different tumour characteristics within *BRCA1 *and *BRCA2 *mutation carriers.

As an adjunct to predictive testing for a high risk *BRCA1 *or *BRCA2 *gene mutation, more individualized risk estimates that take into account additional genetic and environmental modifiers will require a more detailed understanding of how these various risk factors interact. Understanding whether common genetic variants modify the risks of developing ER-positive or ER-negative breast cancer in *BRCA1 *and *BRCA2 *mutation carriers could potentially influence the clinical management of these individuals. For example, knowing that a *BRCA1 *mutation carrier is more likely to develop ER-positive breast cancer (than most *BRCA1 *mutation carriers), may influence the choice of management strategies, such as chemoprevention. In this study, we used data from the CIMBA consortium to evaluate the associations between the 12 common breast cancer susceptibility alleles and risk for breast cancer defined by ER and PR status.

## Materials and methods

### Subjects

Subjects were *BRCA1 *and *BRCA2 *mutation carriers recruited by 36 study centres in Europe, North America and Australia (Table 1). All carriers participated in clinical or research studies at the host institutions, which have been approved by local ethics committees (list provided in Additional file 1, Table S1). Each committee granted approval for access and use of the medical records for the present analyses.

**Table 1:** Number of mutation carriers by country grouping affection status and tumour marker characteristics

Country Group	BRCA1	BRCA2	Unaffected	Breast Cancer	ER-	ER+	PR-	PR+
								
Austria^1^	465	179	318	326	76	51	76	44
Australia^2^	660	552	541	671	235	200	297	121
Canada^3^	443	358	386	415	107	70	89	68
Denmark^4^	507	319	463	363	98	93	79	45
France-Belgium-Spain^5^	1,673	1,256	1,217	1,712	140	165	1,661	127
Finland^6^	103	105	91	117	59	54	74	39
Germany^7^	1,231	589	648	1,172	443	336	457	311
Iceland^8^	0	135	24	111	21	57	18	57
Italy^9^	994	666	686	974	203	251	231	216
Latvia-Lithuania-Russia^10^	190	0	79	111	21	6	18	7
Sweden^11^	537	177	396	318	86	54	89	50
Netherlands^12^	804	319	611	512	72	41	69	29
UK-Eire^13^	1,107	866	1,008	965	268	239	175	104
USA^14^	2,707	1559	2,118	2048	482	366	512	297
								
Total	11,421	7,080	8,686	9,815	2,311	1,983	2,345	1,515

Studies in country groups:1:MUV; 2:BCFR/KCONFAB; 3:OCGN/BCFR/INHERIT; 4:CBCS/OUH; 5: GEMO/CNIO/HCSC/ICO/MOD-SQUAD; 6:HEBCS; 7:GC-HBOC/DKFZ; 8:ILUH; 9:CONSIT-TEAM/IOVHBOCS/PBCS; 10: NNPIO/BFBOCC; 11: SWE-BRCA; 12: HEBON; 13:EMBRACE/UKGRFOCR; 14:FCCC/GEMO/GEORGETOWN/MAGIC/MAYO/MSKCC/NCI/OSU-CCG/UCI/UCSF/UKGRFOCR/UPENN/WCRI

The large majority of carriers were recruited through cancer genetics clinics offering genetic testing, and enrolled into national or regional studies. Eligibility to participate in CIMBA is restricted to female carriers of pathogenic *BRCA1 *or *BRCA2 *mutations who were 18 years old or older at recruitment. Information collected included the year of birth; mutation description, including nucleotide position and base change; age at last follow-up; ages at breast and ovarian cancer diagnoses; and age or date at bilateral prophylactic mastectomy. Information was also available on the country of residence. Related individuals were identified through a unique family identifier. Women were included in the analysis if they carried mutations that were pathogenic according to generally recognized criteria. Only studies that provided tumour pathology information and had genotype information were included in the analysis. However, to maximise the available information, genotyped mutation carriers within those studies missing information on tumour characteristics were included in the analysis and their disease subtype was assumed to be missing at random (see statistical methods for details). Further details about the CIMBA initiative can be found elsewhere [33].

### Tumour pathology data collection

Tumour pathology data were amalgamated from a range of sources, specifically patient pathology reports, medical records, pathology review data, tumour registry records and results from tissue microarrays. Estrogen and progesterone receptor status was provided as negative or positive, with supplementary immunohistochemistry scoring data and methodology provided when available. Based on definitions supplied, most centres employed a cut off of ≥10% of tumour cells stained positive to define receptor positivity. To ensure consistency across studies, when information on the proportion of cells stained was available, we used the same cut-off to define ER and PR positive tumours. For a small number of cases where composite scoring methods based on the proportion and intensity of staining were available (Allred score, Remmele score and H-score), widely-accepted cut-offs were used (Additional file 1, Table S2). Consistency checks were performed to validate receptor data against supplementary scoring information if provided.

### Genotyping

This analysis included genotype data on 12 SNPs that had been previously assessed for their associations with the overall risk of breast cancer for *BRCA1 *and *BRCA2 *mutation carriers in CIMBA. Genotyping was performed using either the iPLEX or Taqman platforms and has been described in detail in the previous reports [28-31]. To ensure genotyping consistency, all genotyping centres were required to adhere to the CIMBA genotyping quality control criteria which are described in detail online [34]. The 12 SNPs genotyped were rs2981582 in *FGFR2*, rs3803662 in *TOX3/TNRC9*, rs889312 in *MAP3K1*, rs3817198 in *LSP1*, rs13387042 at 2q35, rs13281615 at 8q24, rs4973768 near *SLC4A7/NEK10*, rs6504950 in the *STXBP4/COX11 *region, rs2046210 near *ESR1 *at 6q25.1 and rs11249433 at 1p11.2. A Taqman assay could not be adequately designed for SNP rs999737 in the *RAD51L1 *region and studies using this platform genotyped the surrogate SNP rs10483813 (pair-wise r^2 ^= 1 with rs999737 based on HapMap CEU data). Data for these two SNPs were combined and treated as a single locus in the analysis of associations.

### Statistical analysis

The aim of this study was to evaluate the associations between each genotype and breast cancer subtypes defined by tumour characteristics in *BRCA1 *and *BRCA2 *mutation carriers separately. The phenotype of each individual was defined by the age at diagnosis of breast cancer and its subtype or by age at last follow-up. Individuals were censored at the age of the first breast cancer diagnosis, ovarian cancer diagnosis, or bilateral prophylactic mastectomy or the age at last observation. Mutation carriers censored at ovarian cancer diagnosis were considered unaffected.

The analysis of risk modifiers in *BRCA1 *and *BRCA2 *mutation carriers is complicated by the fact that mutation carriers are not randomly sampled with respect to their disease status. Many carriers are sampled through families seen in genetic clinics. The first tested individual in a family is usually someone diagnosed with cancer at a relatively young age. Such study designs, therefore, tend to lead to an over-sampling of affected individuals, and standard analytical methods like Cox regression or case-control analysis may lead to biased estimates of the risk ratios [35]. This can be illustrated by considering an individual affected at age *t*. In a standard analysis of a cohort study or a case-control analysis, the SNP genotype for the individual will be compared with those of all individuals at risk at age *t *or in a case-control analysis, with controls randomly sampled from all possible at risk individuals. This analysis leads to consistent estimates of the hazard ratio or odds ratio estimates. However, in the present design, mutation carriers are already selected on the basis of disease status (where affected individuals are over-sampled). If standard cohort analysis were applied to these data, it would lead to affected individuals at age *t *being compared to unaffected carriers selected on the basis of their future disease status. If the genotype is associated with the disease, the risk estimate will be biased to zero because too many affected individuals (in whom the at-risk genotype is overrepresented) are included in the comparison group. Simulation studies have shown that this effect can be quite marked [35]. To address this, a retrospective likelihood approach was previously proposed, which models the observed genotypes conditional on the disease phenotypes [36]. For the current analyses we have extended this method to model the simultaneous effect of each SNP on more than one tumour subtype. We briefly describe this method for the analysis of associations with ER-positive and ER-negative breast cancer, but the same principles apply for the analysis of associations with other tumour characteristics.

We modelled the likelihood of the observed genotypes and tumour subtype conditional on the disease status, that is:

(1) $$\overset{n}{\underset{i=1}{\prod }}P\left(g_{i},d_{i}|y\left(t_{i}\right)\right)=\overset{n}{\underset{i}{\prod }}\frac{P\left(\left(y\left(t_{i}\right),d_{i}|g_{i}\right)\right)P\left(g_{i}\right)}{P\left(y\left(t_{i}\right)\right)}$$

Where *y*(*t_i_*) is the disease phenotype for individual *i *at censoring age *t_i _*(breast cancer at age *t_i _*or unaffected at age *t_i_*), *d_i _*is the ER status (0 = negative, 1 = positive) and *g_i _*the observed genotype of individual *i *(*g_i _*= 0, 1 or 2 minor alleles) and *n *the number of subjects in the analysis. To allow for tumour characteristics we assumed that breast cancer consists of different disease subtypes, such that the total breast cancer incidence at age *t_i_*, *λ*(*t_i_*), is the sum of the disease incidence for the subtypes, that is *λ*(*t_i_*) = *v*(*t_i_*) + *μ*(*t_i_*), where *v*(*t_i_*) is the incidence for ER-negative disease and *μ*(*t_i_*) is the incidence of ER-positive disease. We assumed that the subtype-specific incidences depend on the underlying genotype through a Cox-proportional hazards model: $\nu \left(t_{i}\right)=\nu_{0}\left(t_{i}\right)\exp\left(\beta^{t}z_{g_{i}}\right)$ and $\mu \left(t_{i}\right)=\mu_{0}\left(t_{i}\right)\exp\left(\gamma^{t}z_{g_{i}}\right)$where *v*_0_(*t_i_*) and *μ*_0_(*t_i_*) and are the baseline incidences for disease subtypes (ER-negative and ER-positive respectively), $z_{g_{i}}$is the genotype vector for individual *i *and ***β ***and ***γ ***are the subtype specific genotype log-risk ratios (for ER-negative and ER-positive breast cancer respectively). The probabilities of developing ER-positive and ER-negative breast cancer conditional on the underlying genotype were assumed to be independent. We further assumed that, if tumour subtype is unknown, the information is missing at random with respect to genotype. Then for each individual:

$$P\left(\left(y\left(t_{i}\right),d_{i}|g_{i}\right)\right)=\begin{array}{c} \begin{array}{c} ={\left(\nu_{0}\left(t_{i}\right)\exp\left(\beta^{t}z_{g_{i}}\right)\right)}^{O_{i}\left(1-d_{i}\right)}\times {\left(\mu_{0}\left(t_{i}\right)\exp\left(\gamma^{t}z_{g_{i}}\right)\right)}^{O_{i}d_{i}}\times \\ \exp\left(-\overset{t_{i}-1}{\underset{u=0}{\sum }}\left(\mu_{0}\left(u\right)\exp\left(\gamma^{t}z_{g_{i}}\right)+\nu_{0}\left(u\right)\exp\left(\beta^{t}z_{g_{i}}\right)\right)\right)\text{ if }d_{i}=0,\text{ 1 } \end{array}\\ \begin{array}{c} \;={\left(\mu_{0}\left(t_{i}\right)\exp\left(\gamma^{t}z_{g_{i}}\right)+\nu_{0}\left(t\right)\exp\left(\beta^{t}z_{g_{i}}\right)\right)}^{O_{i}}\times \\ \exp\left(-\overset{t_{i}-1}{\underset{u=0}{\sum }}\left(\mu_{0}\left(u\right)\exp\left(\gamma^{t}z_{g_{i}}\right)+\nu_{0}\left(u\right)\exp\left(\beta^{t}z_{g_{i}}\right)\right)\right)\text{ if }d_{i}=\text{ unknown} \end{array} \end{array}$$

were *O_i _*= 0 if unaffected and *O_i _*= 1 if affected. Thus, the above formulation allows use of all mutation carriers irrespective of whether the tumour subtype is observed or not. The baseline incidences for each disease subtype (*v_0_*(*t_i_*) and *μ*_0_(*t_i_*)) are unknown. However, it is possible to solve for those recursively by constraining the overall breast cancer incidence for mutation carriers *λ*(*t*), to agree with external estimates as previously demonstrated [37,38] and by imposing a further constraint on the ratio of the observed ER-positive to ER-negative breast cancers in each age group:

The likelihood in equation 1 can then be maximised jointly over the log-risk ratios ***β ***and ***γ***, genotype frequencies *P(g) *and the age and subtype-specific frequencies *π*_+_(*t*) and *π*_-_(*t*) This likelihood is based on the assumption that the ascertainment of mutation carriers is dependent on the overall disease phenotype (breast cancer) but not on tumour subtypes. This allows the subtype frequencies *π*_+_(*t*) and *π_-_*(*t*) to be estimated within the dataset. Relaxing this assumption and conditioning also on tumour subtype requires external estimates for the age and subtype-specific frequencies *π*_+_(*t*) and *π*_-_(*t*).

The effect of each SNP was modelled either as a per-allele HR (multiplicative model) or as separate HRs for heterozygotes and homozygotes, and these were estimated on the logarithmic scale. Heterogeneity in the hazard ratios between tumour subtypes was examined by fitting models where *v*(*t_i_*) = *v*_0_(*t_i_*) exp (*β*_1*g*_) and *μ*(*t_i_*) = *μ*_0_(*t_i_*) exp (*β*_1 _+ *β*_2_)*g*) with g = 0,1 and 2 (for 0, 1, 2 copies of the minor allele respectively) and testing for *β*_2 _= 0. Analyses were carried out with the pedigree-analysis software MENDEL [39]. All analyses were stratified by country of residence and used calendar-year- and cohort-specific cancer incidences for *BRCA1 *and *BRCA2 *[40]. For this purpose, a stratified version of the retrospective likelihood (equation 1) was derived as described previously [36]. Countries with small numbers of mutation carriers were grouped together. We used a robust variance-estimation approach to allow for the non-independence among related mutation carriers [41].

### Predicted breast cancer risks by ER status

Based on our results we computed the predicted absolute risk of developing ER-negative and ER-positive breast cancer for *BRCA1 *and *BRCA2 *mutation carriers by the combined 12 SNP profile. For each individual we derived an empirical score, based on the per-allele log-relative hazard estimates for each genotype, which was of the form $\overset{12}{\underset{j=1}{\sum }}\beta_{j}g_{j}$where *β_j _*is the per-allele log-hazard estimate for locus j and *g_j _*is the genotype at the same locus (taking values 0, 1 and 2). This assumes a multiplicative model for the combined SNP associations. This is a reasonable assumption given that previous analyses found no evidence of departure from the multiplicative model [35]. Scores were calculated for ER-positive and ER-negative disease, separately for *BRCA1*and *BRCA2 *mutation carriers. The empirical distribution of the derived score was then used to compute the subtype specific incidence associated with each multilocus genotype as described previously [31]. We reported the absolute risks of developing ER-specific breast cancer at the 5^th^, 50^th ^and 95^th ^percentiles of the empirical distribution of the SNP profile.

## Results

A total of 11,421 *BRCA1 *and 7,080 *BRCA2 *mutation carriers from 36 studies had been successfully genotyped for at least one of the12 SNPs and were eligible for inclusion in these analyses. 9,815 *BRCA1 *and *BRCA2 *mutation carriers were censored at a first invasive breast cancer diagnosis, of whom 4,310 had information on either ER or PR (Table 1).

### Associations with ER status - BRCA1 mutation carriers

There were significant differences in the HR for ER-positive and ER-negative disease for *BRCA1 *mutation carriers for two SNPs (Table 2). The *FGFR2 *SNP rs2981582 exhibited the clearest difference with a strong association for ER-positive disease but not ER-negative disease (per allele HR = 1.35, 95% CI: 1.17 to 1.56, for ER-positive compared with HR = 0.91, 95% CI: 0.85 to 0.98 for ER-negative, *P*-heterogeneity = 6.5 × 10^-6^). The *SLC4A7/NEK10 *SNP rs4973768 also exhibited a similar pattern (ER-positive: per-allele HR = 1.17, 95% CI: 1.03 to 1.133, compared with ER-negative: per-allele HR = 0.99, 95% CI: 0.93 to 1.06, *P*-heterogeneity = 0.027). Although there was no significant evidence of differences between the HRs for ER-positive and ER-negative breast cancer, the *TOX3/TNRC9 *SNP rs3803662 was significantly associated with the risk of ER-positive disease (per-allele HR = 1.25, 95% CI: 1.06 to .46, *P*-trend = 0.0062) but not with risk for ER-negative breast cancer (per-allele HR = 1.05, 95% CI: 0.97 to 1.13, *P*-trend = 0.21). *LSP1 *SNP rs3817198 was associated with the risk of ER-negative breast cancer (per-allele HR = 1.07, 95% CI: 1.00 to 1.07, *P*-trend = 0.047) but not with risk of ER-positive breast cancer (per-allele HR = 1.07, 95% CI: 0.93 to 1.22, *P*-trend = 0.33, *P*-het = 0.98). The 6q25.1 SNP rs2046210 near *ESR1 *was associated with the risk for both ER-negative (per-allele HR = 1.19, 95% CI: 1.11 to 1.27, *P*-trend = 2.4 × 10^-7^) and ER-positive (per-allele HR = 1.14, 95% CI: 1.01 to 1.30, *P*-trend = 0.043) breast cancer. There was no significant evidence of association with either ER-negative or ER-positive breast cancer for any of the other SNPs, although the HR estimates tended to be higher for ER-positive breast cancer (for example, SNPs rs13387042 at 2q35 and rs13281615 at 8q24).

**Table 2:** Genotype and per-allele hazard ratio estimates by estrogen receptor status for *BRCA1 *mutation carriers

Genotype	**Unaffected**,	Affected by subtype, N (%)			ER-			ER+			*P*-het
	N (%)	ER-	ER+	Unknown	HR	95% CI	*P-trend*	HR	95% CI	*P-trend*	
*FGFR2 rs2981582*											
GG	1301 (36.2)	447 (40.0)	104 (29.6)	869 (35.1)	**1.00**			**1.00**			
GA	1,721 (47.9)	516 (46.2)	166 (47.3)	1190 (48.1)	**0.93**	**0.83 to 1.03**		**1.24**	**0.98 to 1.57**		
AA	573 (15.9)	154 (13.8)	81 (23.1)	416 (16.8)	**0.82**	**0.70 to 0.96**		**1.85**	**1.40 to 2.44**		
Per-allele					**0.91**	**0.85 to 0.98**	**0.01**	**1.35**	**1.17 to 1.56**	**4 × 10^-5^**	**6.5 ×10^-6^**
*TOX3/TNRC9 rs3803662*											
CC	1,811 (52.0)	545 (49.5)	154 (45.7)	1195 (50.2)	1.00			**1.00**			
CT	1,405 (40.3)	461 (41.8)	143 (42.4)	987 (41.5)	1.06	0.96 to 1.18		**1.20**	**0.97 to 1.50**		
TT	269 (7.7)	96 (8.7)	40 (11.9)	199 (8.4)	1.09	0.90 to 1.31		**1.61**	**1.14 to 2.27**		
Per-allele					1.05	0.97 to 1.13	0.21	**1.25**	**1.06 to 1.46**	**0.0062**	0.07
*MAP3K1 *rs889312											
AA	1,858 (49.6)	569 (49.7)	186 (52.1)	1319 (51.6)	1.00			1.00			
AC	1,552 (41.4)	480 (41.9)	136 (38.1)	987 (38.6)	0.97	0.87 to 1.07		0.86	0.69 to 1.06		
CC	336 (9.0)	97 (8.5)	35 (9.8)	250 (9.8)	0.97	0.81 to 1.16		0.97	0.69 to 1.35		
Per-allele					0.98	0.91 to 1.06	0.56	0.97	0.83 to 1.13	0.69	0.92
*LSP1 *rs3817198											
TT	1,894 (47.4)	652 (45.4)	195 (44.5)	1205 (43.7)	1.00			1.00			
TC	1,680 (42.0)	629 (43.8)	197 (45.0)	1239 (44.9)	1.09	0.99 to 1.20		1.11	0.92 to 1.35		
CC	422 (10.6)	154 (10.7)	46 (10.5)	315 (11.4)	1.13	0.97 to 1.33		1.10	0.80 to 1.50		
Per-allele					**1.07**	**1.00 to 1.15**	**0.047**	1.07	0.93 to 1.22	0.33	0.98
2q35 rs13387042											
GG	924 (24.0)	301 (22.1)	93 (22.2)	576 (21.4)	1.00			1.00			
GA	1,855 (48.3)	723 (53.1)	194 (46.3)	1370 (50.9)	**1.17**	**1.04 to 1.32**		1.02	0.81 to 1.30		
AA	1,064 (27.7)	338 (24.8)	132 (31.5)	745 (27.7)	0.96	0.84 to 1.11		1.25	0.97 to 1.62		
Per-allele					0.98	0.91 to 1.04	0.48	1.13	0.99 to 1.28	0.075	0.065
8q24 rs13281615											
AA	1,319 (32.8)	502 (35.9)	143 (33.7)	897 (32.5)	1.00			1.0			
AG	2,008 (50.0)	657 (47.0)	198 (46.7)	1,364 (49.5)	0.98	0.88 to 1.08		0.97	0.79 to 1.19		
GG	691 (17.9)	238 (17.0)	83 (19.6)	496 (18.0)	1.00	0.87 to 1.16		1.17	0.89 to 1.53		
Per-allele					1.00	0.93 to 1.07	0.93	1.06	0.93 to 1.22	0.38	0.43
SLC4A7/NEK10 rs4973768											
CC	1,148 (26.2)	406 (27.2)	103 (22.2)	691 (24.7)	1.00			1.00			
CT	2,205 (50.4)	736 (49.3)	235 (50.5)	1,399 (50.1)	0.98	0.88 to 1.08		1.20	0.96 to 1.51		
TT	1,024 (23.4)	350 (23.5)	127 (27.3)	703 (25.2)	0.99	0.87 to 1.12		**1.38**	**1.07 to 1.77**		
Per-allele					0.99	0.93 to 1.06	0.83	**1.17**	**1.03 to 1.33**	**0.013**	**0.027**
STXBP4/COX11 rs6504950											
GG	2,346 (53.1)	814 (53.2)	252 (52.9)	1,502 (53.1))	1.00			1.00			
GA	1,737 (39.3)	593 (37.8)	191 (40.1)	1,127 (39.8)	1.00	0.91 to 1.10		1.03	0.86 to 1.24		
AA	333 (7.5)	122 (8.0)	33 (6.9)	200 (7.1)	1.04	0.88 to 1.23		0.94	0.65 to 1.34		
Per-allele					1.01	0.94 to 1.09	0.77	1.00	0.87 to 1.15	0.97	0.87
5p12 rs10941679											
AA	2,211 (55.8)	815 (57.3)	271 (61.0)	1,529 (56.5)	1.00			1.00			
AG	1,472 (37.1)	517 (36.4)	145 (32.7)	1,001 (37.0)	0.99	0.90 to 1.09		0.84	0.69 to 1.02		
GG	280 (7.1)	90 (6.3)	28 (6.3)	177 (6.5)	0.89	0.73 to 1.08		0.84	0.58 to 1.20		
Per-allele					0.97	0.90 to 1.04	0.39	0.88	0.75 to 1.02	0.08	0.26
6q25.1 - rs2046210											
CC	1,886 (43.3)	567 (38.2)	158 (36.0)	1,007 (37.0)	**1.00**			**1.00**			
TC	1,919 (44.1)	718 (48.3)	232 (52.9)	1,305 (48.0)	**1.21**	**1.10 to 1.33**		**1.37**	**1.13 to 1.67**		
TT	547 (12.6)	201 (13.5)	49 (11.2)	409 (15.0)	**1.39**	**1.21 to 1.59**		**1.11**	**0.81 to 1.53**		
Per-allele					**1.19**	**1.11 to 1.27**	**2.4 × 10^-7^**	**1.14**	**1.01 to 1.30**	**0.043**	0.60
1p11.2 - rs11249433											
TT	1,491 (34.1)	506 (33.7)	144 (32.4)	973 (35.1)	1.00			1.00			
CT	2,133 (48.7)	745 (49.7)	245 (55.2)	1,342 (48.5)	0.97	0.88 to 1.07		1.10	0.90 to 1.34		
CC	752 (17.2)	248 (16.5)	55 (12.4)	455 (16.4)	0.99	0.87 to 1.13		0.76	0.56 to 1.03		
Per-allele					0.99	0.93 to 1.06	0.79	0.92	0.81 to 1.05	0.22	0.35
***RAD51L1 *- rs999737/rs10483813**											
CC/TT	2,335 (61.5)	760 (64.1)	212 (62.5)	1,551 (62.5)	1.00			1.00			
TC/AT	1,294 (34.1)	370 (31.2)	113 (33.3)	819 (33.0)	0.92	0.83 to 1.03		1.00	0.80 to 1.24		
TT/AA	170 (4.5)	56 (4.7)	14 (4.1)	110 (4.4)	1.02	0.81 to 1.28		0.93	0.56 to 1.56		
per allele					0.96	0.88 to 1.04	0.34	0.98	0.82 to 1.18	0.87	0.81

*P*-het, Heterogeneity *P*-value; ER-, estrogen receptor negative; ER+, estrogen receptor positive

### Associations with ER status - BRCA2 mutation carriers

Only SNP rs2046210 at 6q25.1 exhibited differential associations between ER-positive and ER-negative breast cancer for *BRCA2 *mutation carriers (*P*-heterogeneity = 0.045, Table 3). The per-allele HR for ER-negative disease was estimated to be 1.17 (95% CI: 0.99 to 1.38) whereas the per-allele HR for ER-positive breast cancer was 0.97 (95% CI: 0.89 to 1.05). Although there were no significant differences in the associations between the two types of disease for *BRCA2 *mutation carriers, the HR estimates for ER-positive disease tended to be larger compared to ER-negative breast cancer. SNPs at/near *FGFR2, TOX3/TNRC9, MAP3K1, LSP1*, 2q35*, SLC4A7/NEK10*, 5p12 and 1p11.2 were associated with ER-positive breast cancer for *BRCA2 *mutation carriers (using either a per-allele or 2 df genotype test). The strongest associations were for the *FGFR2 *rs2981582 SNP (HR for ER-positive breast cancer = 1.35, 95% CI: 1.23 to 1.48, *P*-trend = 1.4 × 10^-10^) and *TOX3/TNRC9 *SNP rs3803662 (HR for ER-positive breast cancer = 1.28. 95% CI: 1.16 to 1.41, *P*-trend = 1.5 × 10^-6^). Only SNPs at or near *MAP3K1, STXBP4/COX11 *and 6q25.1 were associated with the risk of ER-negative breast cancer for *BRCA2 *mutation carriers.

**Table 3:** Genotype and per-allele hazard ratio estimates by estrogen receptor status for *BRCA2 *mutation carriers

Genotype	**Unaffected**,	Affected by subtype, N (%)			ER-			ER+			*P*-het
	N (%)	ER-	ER+	Unknown	HR	95%CI	*P-trend*	HR	95% CI	*P-trend*	
*FGFR2 rs2981582*											
GG	794 (37.8)	86 (32.7)	248 (29.5)	457 (29.8)	1.00			**1.00**			
GA	987 (47.0)	137 (52.1)	419 (49.8)	755 (49.3)	1.28	0.99 to 1.67		**1.35**	**1.17 to 1.55**		
AA	321 (15.3)	40 (15.2)	174 (20.7)	320 (20.9)	1.23	0.85 to 1.78		**1.81**	**1.51 to 2.18**		
Per-allele					1.14	0.97 to 1.35	0.12	**1.35**	**1.23 to 1.48**	**1.4 × 10^-10^**	0.097
*TOX3/TNRC9 rs3803662*											
CC	1,088 (53.4)	136 (53.3)	377 (46.3)	702 (48.2)	1.00			**1.00**			
CT	792 (38.9)	96 (37.7)	361 (44.3)	604 (41.5)	0.98	0.75 to 1.27		**1.33**	**1.17 to 1.53**		
TT	157 (7.7)	23 (9.0)	77 (9.5)	150 (10.3)	1.27	0.83 to 1.93		**1.54**	**1.22 to 1.95**		
Per-allele					1.06	0.88 to 1.29	0.53	**1.28**	**1.16 to 1.41**	**1.5 × 10^-6^**	0.11
*MAP3K1 *rs889312											
AA	1,107 (51.1)	121 (45.7)	430 (50.3)	746 (47.7)	1.00			1.00			
AC	888 (41.0)	120 (45.3)	349 (40.8)	646 (41.3)	1.23	0.96 to 1.59		1.03	0.90 to 1.17		
CC	170 (7.9)	24 (9.1)	76 (8.9)	172 (11.0)	1.42	0.93 to 2.16		**1.29**	**1.03 to 1.62**		
Per-allele					**1.21**	**1.01 to 1.45**	**0.039**	1.09	0.99 to 1.21	0.08	0.35
*LSP1 *rs3817198											
TT	1,075 (46.1)	142 (44.4)	429 (42.0)	718 (42.7)	1.00			**1.00**			
TC	1,005 (43.1)	146 (45.6)	466 (45.6)	759 (45.2)	1.08	0.86 to 1.36		**1.14**	**1.01 to 1.29**		
CC	252 (10.8)	32 (10.0)	127 (12.4)	203 (12.1)	1.02	0.68 to 1.51		**1.39**	**1.14 to 1.70**		
Per-allele					1.03	0.87 to 1.22	0.70	**1.17**	**1.07 to 1.28**	**5.5 × 10^-4^**	0.20
2q35 rs13387042											
GG	571 (25.3)	71 (23.0)	216 (22.0)	382 (23.1)	1.00			1.00			
GA	1,080 (47.8)	156 (50.5)	500 (50.8)	809 (48.8)	1.12	0.85 to 1.47		**1.18**	**1.01 to 1.36**		
AA	608 (26.9)	82 (26.5)	268 (27.2)	466 (28.1)	1.06	0.78 to 1.45		1.13	0.95 to 1.34		
Per-allele					1.03	0.87 to 1.19	0.71	1.06	0.97 to 1.15	0.20	0.75
8q24 rs13281615											
AA	794 (34.1)	99 (31.6)	317 (31.7)	524 (31.3)	1.00			1.00			
AG	1,156 (49.6)	165 (52.7)	511 (51.1)	837 (49.9)	1.08	0.85 to 1.38		1.05	0.92 to 1.21		
GG	382 (16.4)	49 (15.7)	172 (17.2)	315 (18.8)	1.05	0.75 to 1.46		1.13	0.94 to 1.35		
Per-allele					1.04	0.89 to 1.21	0.66	1.06	0.97 to 1.16	0.19	0.80
SLC4A7/NEK10 rs4973768											
CC	669 (26.5)	82 (24.9)	251 (22.6)	401 (23.5)	1.00			**1.00**			
CT	1,241 (49.1)	164 (49.9)	546 (49.4)	829 (48.7)	1.05	0.81 to 1.36		**1.14**	**0.99 to 1.31**		
TT	618 (24.5)	83 (25.2)	311 (28.0)	474 (27.8)	1.04	0.77 to 1.41		**1.27**	**1.08 to 1.50**		
Per-allele					1.02	0.88 to 1.19	0.78	**1.13**	**1.04 to 1.22**	**0.0043**	0.25
STXBP4/COX11 rs6504950											
GG	1,420 (55.6)	171 (51.0)	601 (53.1)	896 (52.5)	1.00			1.00			
GA	951 (37.2)	145 (43.3)	444 (39.3)	684 (40.1)	**1.27**	**1.03 to 1.58**		1.09	0.97 to 1.23		
AA	184 (7.2)	19 (5.7)	86 (7.6)	127 (7.4)	0.84	0.54 to 1.30		1.08	0.87 to 1.34		
Per-allele					1.07	0.92 to 1.25	0.36	1.06	0.97 to 1.16	0.19	0.91
5p12 rs10941679											
AA	1,372 (58.6)	176 (54.8)	584 (54.8)	924 (56.3)	1.00			1.00			
AG	824 (35.2)	122 (38.0)	425 (39.9)	622 (37.9)	1.08	0.86 to 1.37		**1.15**	**1.01 to 1.30**		
GG	146 (6.2)	23 (7.2)	57 (5.4)	94 (5.7)	1.25	0.82 to 1.91		0.94	0.73 to 1.22		
Per-allele					1.10	0.92 to 1.31	0.28	1.06	0.96 to 1.17	0.23	0.70
6q25.1 - rs2046210											
CC	985 (39.8)	121 (39.2)	466 (42.1)	634 (37.7)	**1.00**			1.00			
TC	1,165 (47.1)	132 (42.7)	499 (45.1)	802 (47.7)	**0.99**	**0.78 to 1.12**		0.96	0.85 to 1.08		
TT	324 (13.1)	56 (18.1)	141 (12.8)	247 (14.7)	**1.47**	**1.08 to 2.01**		0.94	0.78 to 1.12		
Per-allele					1.17	0.99 to 1.38	0.059	0.97	0.89 to 1.05	0.41	**0.045**
1p11.2 - rs11249433											
TT	895 (35.9)	107 (33.6)	345 (31.4)	599 (34.7)	1.00			**1.00**			
CT	1,226 (49.2)	160 (50.3)	553 (50.3)	843 (48.9)	1.00	0.79 to 1.28		**1.08**	**0.96 to 1.23**		
CC	371 (14.9)	51 (16.0)	202 (18.4)	282 (16.4)	1.00	0.73 to 1.40		**1.27**	**1.08 to 1.50**		
Per-allele					1.00	0.86 to 1.17	0.98	**1.12**	**1.03 to 1.22**	**0.0065**	0.23
***RAD51L1 *- rs999737/rs10483813**											
*BRCA1*	CC/TT	1,368 (59.5)	167 (59.0)	589 (61.4)	1,000 (62.0)	1.00			1.00		
TC/AT	789 (34.3)	104 (36.8)	323 (33.6)	534 (33.1)	1.06	0.84 to 1.35		0.94	0.83 to 1.07		
TT/AA	141 (6.1)	12 (4.2)	48 (5.0)	80 (4.9)	0.74	0.42 to 1.32		0.87	0.67 to 1.14		
Per-allele					0.97	0.80 to 1.17	0.73	0.94	0.85 to 1.03	0.20	0.77

*P*-het, Heterogeneity *P*-value; ER-, estrogen receptor negative; ER+, estrogen receptor positive

### Associations with PR status - BRCA1 mutation carriers

The general pattern of associations with PR-positive and PR-negative breast cancer for *BRCA1 *mutation carriers (Additional file 1, Table S3) was similar to that seen for ER status. Significant differences in the associations between PR-positive and PR-negative breast cancer were observed for three SNPs. The minor allele of *FGFR2 *SNP rs2981582 was associated with a significantly higher risk for PR-positive breast cancer for *BRCA1 *mutation carriers (per-allele HR for PR-positive = 1.29, 95% CI: 1.10 to 1.51, HR for PR-negative = 0.93, 95% CI: 0.87 to 1.00, P-heterogeneity = 7 × 10^-4^). Allele "A" of SNP rs13387042 at 2q35 was associated with a significantly higher risk of PR-positive breast cancer for *BRCA1 *mutation carriers (HR for PR-positive breast cancer = 1.16, 95% CI: 1.01 to 1.33; HR for PR-negative = 0.97, 95% CI: 0.91 to 1.04, *P*-heterogeneity = 0.034). Although the *RAD51L1 *SNP showed no differential associations with ER-status, there was evidence that the minor allele of this SNP was associated with a lower risk of PR-positive breast cancer (HR for PR-positive = 0.79, 95% CI: 0.61 to 0.95; HR for PR-negative = 1.02, 95% CI: 0.94 to 1.11; *P*-heterogeneity = 0.027). The only SNPs associated with risk for PR-negative breast cancer were SNPs at 6q25.1 (per-allele HR = 1.19, 95% CI: 1.11 to 1.27, *P*-trend = 3.7 × 10^-7^), and in *LSP1 *(per-allele HR = 1.09, 95% CI: 1.01 to 1.16, *P*-trend = 0.017), but these were not significantly different from the associations with PR-positive breast cancer for *BRCA1 *mutation carriers.

### Associations with PR status - BRCA2 mutation carriers

Only two SNPs demonstrated significant differences in the associations with PR-positive and PR-negative breast cancer for *BRCA2 *mutation carriers (Additional file 1, Table S4). SNP rs13387042 at 2q35 was associated with a higher risk of PR-positive breast cancer (per-allele HR for PR-positive = 1.14, 95% CI: 1.03 to 1.25; per-allele HR for PR-negative = 0.92, 95% CI: 0.81 to 1.04, *P*-heterogeneity = 0.009). SNP rs10941679 at 5p12 was also associated with a higher risk of PR-positive breast cancer for *BRCA2 *mutation carriers (per-allele HR for PR-positive = 1.15. 95% CI: 1.03 to 1.27, HR for PR-negative = 0.94, 95% CI: 0.81 to 1.09, *P*-heterogeneity = 0.028). SNPs near or at *FGFR2, TOX3/TNRC9, LSP1 *were associated with both PR-negative and PR-positive breast cancer, whereas the 1p11.2 SNP was associated with risk for PR-negative breast cancer. *MAP3K1 *and *SLC4A7/NEK10 *were associated only with risk of PR-positive breast cancer among *BRCA2 *mutation carriers.

### Absolute risks of developing ER-positive and ER-negative breast cancer by SNP profile

**Figure 1:**
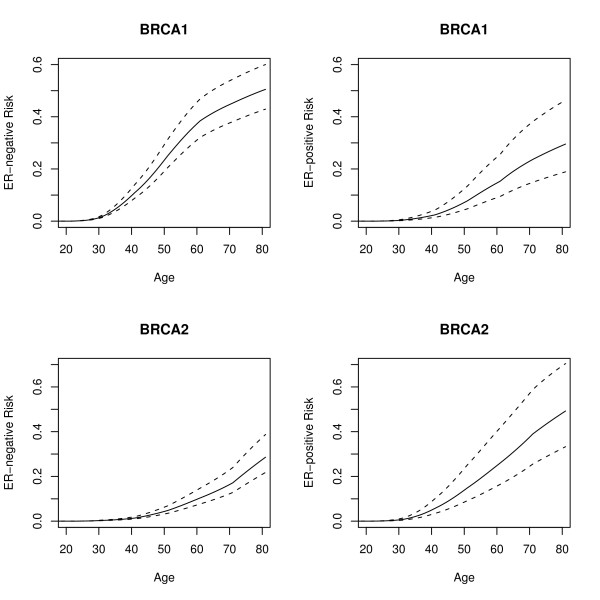
**Predicted risks of developing ER-negative and ER-positive breast cancer based on SNP profiles**. Solid lines depict the median risks and dotted lines the risks at the 5^th ^and 95^th ^percentiles of the risk distribution. The absolute risk differences between individuals at the extremes of the risk distributions are greater for ER-positive breast cancer.

Using the estimated HRs for ER-positive and ER-negative breast cancer for *BRCA1 *and *BRCA2 *mutation carriers, we computed the predicted absolute risk of developing ER-negative and ER-positive breast cancer at various percentiles of the combined SNP distribution. The SNP profile distribution is different for each disease subtype and mutation. We note that SNPs for which the per-allele HR estimates are close to 1.0 contribute little to the predicted ER-specific risks. Figure 1 shows the predicted risks of developing ER-negative and ER-positive breast cancer for *BRCA1 *and *BRCA2 *mutation carriers at the 5^th^, 50^th ^and 95^th ^percentiles of the empirical risk distribution of the combined SNP profile. A *BRCA1 *mutation carrier at the 5^th ^percentile of the SNP profile distribution would be at 43% risk of developing ER-negative breast cancer by age 80 compared with 60% for *BRCA1 *mutation carriers at the 95^th ^percentile of the risk distribution. The risks of developing ER-positive breast cancer would be 18% and 46% by age 80 at the 5^th ^and 95^th ^percentiles of the ER-positive breast cancer risk distribution. *BRCA2 *mutation carriers at the 5^th ^percentile of the ER-negative breast cancer risk distribution are predicted to have a 22% risk of developing ER-negative breast cancer by age 80 compared with 39% for the 95^th ^percentile of the risk distribution. The risks of developing ER-positive breast cancer by age 80 for *BRCA2 *carriers varied from 33% to 70% at the 5^th ^and 95^th ^percentiles of the ER-positive risk distribution respectively.

## Discussion

This is the first report to investigate the associations between 12 common breast cancer susceptibility alleles and ER and PR status of breast tumours in *BRCA1 *and *BRCA2 *mutation carriers. The analysis was made possible by the availability of a large, combined dataset with genotype and tumour pathology information in mutation carriers collated through the CIMBA consortium.

**Figure 2:**
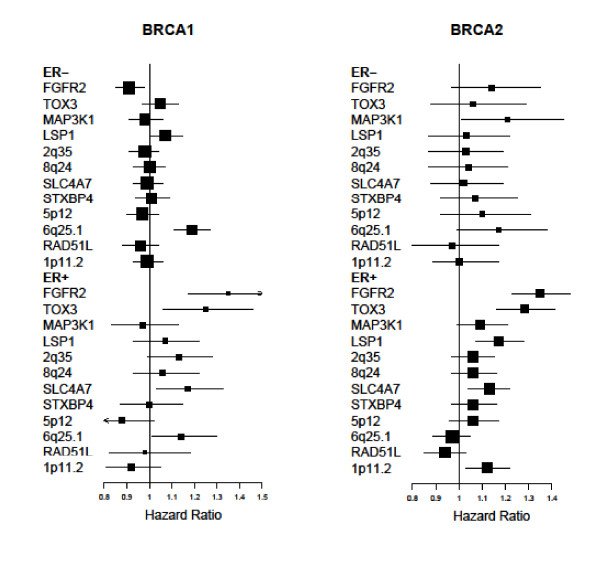
**Summary of per-allele HR estimates for ER-positive and ER-negative breast cancer for mutation carriers**. The patterns of per-allele HR estimates (taken from Tables 2 and 3) suggest that the breast cancer subtype specific associations are similar between *BRCA1 *and *BRCA2 *mutation carriers.

The majority of the SNPs examined demonstrated stronger associations with ER-positive breast cancer for both *BRCA1 *and *BRCA2 *mutation carriers (Figure 2). Only rs2046210 on 6q25.1 exhibited stronger evidence for ER-negative disease. Among *BRCA1 *mutation carriers, the most marked difference was for SNP rs2981582 in *FGFR2*, which was strongly associated with ER-positive breast cancer and exhibited no evidence of an association with ER-negative breast cancer (*P *= 6.5 × 10^-6^). Previous analyses of this polymorphism in mutation carriers failed to find an association with the overall risk of breast cancer for *BRCA1 *mutation carriers, but found an association with risk for *BRCA2 *mutation carriers [29,31]. Our results suggest that rs2981582 in *FGFR2 *also modifies ER-positive breast cancer risk for *BRCA1 *mutation carriers to a similar relative extent as in *BRCA2 *mutation carriers and ER-positive disease in the general population [27,32]. Similar patterns were observed for SNPs rs3803662 in *TOX3/TNRC9 *and rs4973768 in *SLC4A7/NEK10 *in which the associations were predominantly with ER-positive breast cancer for both *BRCA1 *and *BRCA2 *mutation carriers, in line with results from studies of breast cancer in the general population [18,21,27]. The HR estimates for ER-positive breast cancer in *BRCA1 *and *BRCA2 *mutation carriers for these SNPs were very similar.

Among the 12 SNPs investigated in this report, SNP rs2046210 at 6q25.1 exhibited the strongest association with the risk of breast cancer for *BRCA1 *mutation carriers in previous analyses, and was not associated with risk for *BRCA2 *mutation carriers [28]. The current results suggest that this was mainly driven by an association with ER-negative breast cancer risk. This observation is again consistent with the effects seen in population-based studies, in which the relative risk is higher for ER-negative than ER-positive disease [42,43] (Alison Dunning, personal communication). There was some evidence that the 6q25.1 SNP is also associated with ER-negative disease cancer subtype in *BRCA2 *mutation carriers, although the estimates for ER-negative breast cancer in *BRCA2 *mutation carriers are imprecise due to the relatively small sample size. In addition to the 12 loci investigated in this report, a recently identified locus at 19p13 also appears to be predominantly associated with ER-negative breast cancer [44].

The patterns of association between the SNPs and PR tumour status were similar to those observed for ER, which is not surprising given that ER and PR expression are highly correlated. There were, however, two notable exceptions. The 2q35 SNP rs13387042 demonstrated significantly stronger associations with PR-positive than PR-negative breast cancer for both *BRCA1 *and *BRCA2 *mutation carriers (*P *= 0.034 and *P *= 0.0086, for PR-positive for *BRCA1 *and *BRCA2 *respectively), suggesting this SNP may be more relevant for *BRCA1 *and *BRCA2 *tumours expressing PR. However, a population-based study has found this SNP is also associated with PR-negative breast cancer [45]. Furthermore, the *RAD51L1 *locus was associated with PR-positive breast cancer for *BRCA1 *mutation carriers and the magnitude of the association was similar to that observed in the general population [23] (A.B. Spurdle, personal communication).

Previous studies demonstrated that SNPs, which are associated with ER-positive breast cancer in the general population, tend to be associated with the breast cancer risk for *BRCA2 *mutation carriers and SNPs, which are associated with ER-negative breast cancer in the general population, tend to be associated with the breast cancer risk for *BRCA1 *mutation carriers [27,31,44-46]. The current results demonstrate that despite lack of an association between a SNP and the overall breast cancer risk for *BRCA1 *or *BRCA2 *mutation carriers, residual associations exist with specific disease subtypes. Figure 2 summarises the association patterns in *BRCA1 *and *BRCA2 *mutation carriers. The HR estimates for ER-positive and ER-negative breast cancer among *BRCA1 *mutation carriers appear to be different (intraclass correlation coefficient (ICC) approximately 0), as are the HR estimates for ER-positive and ER-negative breast cancer among *BRCA2 *mutation carriers (ICC = 0.13). On the other hand the HR estimates for ER-positive breast cancer among *BRCA1 *and *BRCA2 *mutation carriers appear to be more similar (ICC = 0.65). There is, however, little correlation in the HR estimates for ER-negative breast cancer among *BRCA1 *and *BRCA2 *mutation carriers (ICC = 0.05). However, SNP 6q25.1, which is mainly associated with ER-negative disease in *BRCA1 *mutation carriers, is estimated to confer similar HRs for ER-negative breast cancer for both *BRCA1 *and *BRCA2 *mutation carriers. These associations are mainly in the same direction and of similar magnitude to those observed with breast cancer in the general population stratified by ER expression status. Taken together, these findings are consistent with a model in which these SNPs and *BRCA1 *or *BRCA2 *mutations combine multiplicatively on the risk for ER-positive or ER-negative breast cancer [47]. Hence, the apparent differences in the strength of the SNP associations by *BRCA1 *and *BRCA2 *mutation status can be explained once tumour subtype is taken into account.

The major strength of the current study is the large sample of *BRCA1 *and *BRCA2 *mutation carriers with SNP and tumour marker information. Despite the large sample size, ER and PR marker information was only available for approximately 30% of the mutation carriers that had been diagnosed with breast cancer. The sample sizes for tumour subtypes, while still large, were, therefore, much smaller than were available for analyses of breast cancer risk overall, particularly for ER-positive breast cancer in *BRCA1 *carriers and ER-negative breast cancer in *BRCA2 *carriers. However, by analysing the data using a retrospective cohort approach and analysing the associations with ER-positive and ER-negative disease simultaneously we were able to include all mutation carriers in the analysis, including affected individuals with missing ER status, thus maximizing the available information. Ongoing efforts by CIMBA aim to increase the proportion of mutation carriers diagnosed with breast cancer who also have available tumour pathology information. This will enable us to assess the associations with breast cancer subtypes with greater precision.

The majority of the mutation carriers in CIMBA are identified through clinical genetics centers and, therefore, the source of information or definition of tumour marker status could vary across studies. This heterogeneity in classification may attenuate some of the differences by tumour type. For example, most commonly, a cut-off of 10% of cells staining was taken to denote positivity for ER and PR by the centers without further information on intensity or proportion of positive tumour nuclei and this was used for all our analyses; however, in centers that use the Allred score, a value of > 2 denoted positivity, which may reflect as few as 1% of cells staining. In fact, recent recommendations suggest that ER and PgR assays be considered positive, for therapeutic purposes, if there are at least 1% positive tumour nuclei [48], but these data were not available for the majority of carriers in our samples to enable reclassification. It has been shown, however, that ER is almost always diffusely positive or completely negative (that is, it shows a bimodal staining pattern) with few cases falling between these extremes [49]. Given the small number of tumours likely to fall into the1 to 9% of cells staining category, the impact of changing the cutoff to 1% on our results would be limited. Furthermore, there was no evidence of variation in the distributions of ER or PR status across the studies separately for *BRCA1 *and *BRCA2 *tumours (Mavaddat N, Antoniou AC, personal communication, manuscript in preparation) and all analyses were stratified by country. Finally, the clear differences observed for some SNPs (most notably for *FGFR2 *rs2981582, where the association was limited to ER-positive disease) suggest that the effect of misclassification in tumour subtype on the SNP associations is likely to have been small.

*BRCA1 *and *BRCA2 *tumours have also been found to differ in terms of other tumour characteristics compared to breast cancers in the general population. For example, tumours in mutation carriers are more likely to be of higher grade in comparison to breast cancers in the general population. The distribution of grade has been found to vary between ER-positive and ER-negative tumours in both *BRCA1 *and *BRCA2 *mutation carriers (Mavaddat N, Antoniou AC, personal communication, manuscript in preparation). Although the number of carriers with information on grade, ER status and SNPs was too small to permit combined analysis, our results are unlikely to have been influenced after adjusting for tumour grade. Case-only analysis to test for differences in associations between the SNPs and tumour grade (using ordinal logistic regression) revealed no significant associations between any of the SNPs and grade for both *BRCA1 *and *BRCA2 *mutation carriers (*P *> 0.05 for all tests, results not shown).

The analysis was performed within a retrospective cohort approach, by extending the retrospective likelihood approach described previously [36] to model the simultaneous effects on different breast cancer subtypes defined by ER/PR. Under this approach the associations were estimated simultaneously for the tumour subtypes under investigation. This method depends on the assumption that ascertainment of mutation carriers does not depend on tumour subtypes. This is a reasonable assumption since more than 90% of mutation carriers in our sample were recruited prior to 2007, when it was uncommon to use tumour pathology in selecting individuals for *BRCA1 *and *BRCA2 *mutation screening. Furthermore, the results were virtually identical in a case only, logistic regression analysis for testing for differences in the associations with tumour subtypes which included only individuals with known tumour characteristics (results not shown).

The average risks of developing ER-positive and ER-negative breast cancer in both *BRCA1 *and *BRCA2 *mutation carriers are substantially higher compared to the general population [38]. Therefore, in combination, these SNPs lead to much bigger differences in the absolute risk of developing the disease subtypes between the extremes of the combined SNP genotype distributions [50]. Based on the SNP profiles investigated in this report, the absolute risk difference between mutation carriers at the top 5% of the risk distribution compared to the bottom 5% is much greater for ER-positive breast cancer than for ER-negative breast cancer for both *BRCA1 *and *BRCA2 *(Figure 1). Recent GWAS have identified several other common breast cancer susceptibility variants which have not been investigated in *BRCA1 *and *BRCA2 *mutation carriers yet [24,51]. Moreover, ongoing GWAS in *BRCA1 *and *BRCA2 *mutation carriers [44,52] may also identify further modifiers of breast cancer risk for mutation carriers. It will be important to investigate the associations of these variants with different disease subtypes in *BRCA1 *and *BRCA2 *mutation carriers. Currently, it is unusual for the risks of different disease subtypes to be taken into account in the genetic counseling process. However, as more risk modifying variants are identified in the future, provided these have different associations with different disease subtypes in mutation carriers and confer relative risks which are greater (or smaller) than 1, having precise breast cancer subtype risks may be useful for the planning of the clinical management of both *BRCA1 *and *BRCA2 *mutation carriers. For example, knowing that a female *BRCA1 *mutation carrier was primarily at risk of ER-positive breast cancer based on her associated SNP profile (rather than ER-negative breast cancer, as is the case for the majority of cases) might potentially influence the choice of clinical management by screening, chemoprevention or prophylactic surgery.

## Conclusions

In summary, in this report we investigated the associations of common breast cancer polymorphisms with ER and PR status. Our results indicate there are differential associations between these SNPs and the risk of developing ER-positive or ER-negative breast cancer in *BRCA1 *and *BRCA2 *mutation carriers that mirror similar differences seen in the general population. The findings add to our understanding of the biology of tumour development in mutation carriers and as more risk variants are identified in the future they may improve clinical management of these individuals.

## Abbreviations

CIMBA: Consortium of Investigators of Modifiers of BRCA1/2; ER: estrogen receptor; GWAS: genome-wide association studies; HR: hazard ratio; PR: progesterone receptor; SNPs: single nucleotide polymorphisms.

## Competing interests

The authors declare that they have no competing interests.

## Authors' contributions

AMM, ILA, ACA drafted the initial manuscript. ACA developed the analytical methods and performed the statistical analysis. AMM, ACA, ILA, FJC, DB, SMD, DE, HN, SJR, MR, MS, ABS and BW are members of the CIMBA pathology working group and participated in the design of the study. LM and DB are the CIMBA database managers. AL wrote computer programs for the analysis. SH and OMS reviewed, recoded and classified the BRCA1 and BRCA2 mutations in CIMBA. GCT initiated and coordinated CIMBA. DFE participated in the study design and advised on the statistical analysis. RJ, TvOH, FCNi, BE, AO, IMR, MD, JG, MP, JB, PP, SM, BP, DZ, EC, BB, AV, BP, LP, LO, AS, LB, PR, UH, MV, HEJMH, JW, EBGG, MRN, MK, CS, MMATL, RBvdL, TvO, MR, DF, JLJ, DGE, FL, RE, LI, JA, RD, JC, AD, HD, HG, JE, CH, JB, LES, E McC, AM, SP, AG, RKS, KR, CE, AM, IR, NA, DN, CS, HD, DG, KK, SPA, RVM, IS, BF, WH, DS, HG, VCM, FCFC, SM, ML, NBK, AH, PB, DM, JPF, IM, PP, IC, ML, CK, ML, NS, DSL, CI, TC, MH, TH, KA,IB, CL, RBB, PS, MD, JS, MM, ST, EDA, SF, MY, TR, OO, JNW, HTL, PAG, GET, XW, ZF, VSP, NML, CS, KO, RS, MG, JB, NK, CFS, MKT, DGK, AFR, PLM, MHG, EI, FPO, HO, GG, AET, AMG, MT, TAK, UBJ, ABS, MAC, MS, KH, AvW, BA, MSA, PK, YCD, SLN, MB, PDPP, KBM, KLN, BYK, JG, EMJ, MJD, SMB, MCS, JLH, MBT, WC, AFM and DG acquired phenotypic data and DNA samples or performed SNP genotyping. All authors read and approved the final manuscript.

## Supplementary Material

Additional file 1 **Supplementary Tables**. Supplementary Table 1: List of local ethics committees that approved studies. Supplementary Table 2: Methods and thresholds used to define the final marker variables. Supplementary Table 3: Per-allele hazard ratio estimates by progesterone receptor status for *BRCA1 *mutation carriers. Supplementary Table 4: Per-allele hazard ratio estimates by progesterone receptor status for *BRCA2 *mutation carriers. Click here for file
