# Supplementary material for: Common breast cancer susceptibility alleles are associated with tumour subtypes in BRCA1 and BRCA2 mutation carriers: results from the Consortium of Investigators of Modifiers of BRCA1/2
Source: Breast Cancer Res. 2011 Nov 2;13(6):R110. doi: 10.1186/bcr3052 (PMC3326552; doi:10.1186/bcr3052)
Supplement: Additional file 1 — Supplementary Tables. Supplementary Table 1: List of local ethics committees that approved studies. Supplementary Table 2: Methods and thresholds used to define the final marker variables. Supplementary Table 3: Per-allele hazard ratio estimates by progesterone receptor status for BRCA1 mutation carriers. Supplementary Table 4: Per-allele hazard ratio estimates by progesterone receptor status for BRCA2 mutation carriers. [file bcr3052-S1.DOC]

| **Supplementary Table 1**: List of local ethics committees that approved studies. | | |
| --- | --- | --- |
| **Study** | **Country** | **Committee approval** |
| Breast Cancer Family Registry (BCFR) | USA, | Institutional Review Board University of Utah |
| (BCFR - addtional) | Australia | The University of Melbnourne Health Sciences Human Ethics Sub-Committee |
| (BCFR - addtional) | USA | Columbia University Medical Center Institutional Review Board |
| (BCFR - addtional) | USA | Northern Californa Cancer Center Institutional Review Board |
| (BCFR - addtional) | Canada | University Health Network Research Ethics Board |
| (BCFR - addtional) | Canada | Mount Sinai Hospital Research Ethics Board |
| Baltic Familial Breast and Ovarian Cancer Consortium (BFBOCC) | Latvia, Lithuania | Centrālā medicīnas ētikas Komiteja |
| Copenhagen Breast Cancer Study (CBCS) | Denmark | De Videnskabsetiske Komiteer I Region Hovedsladen |
| Spanish National Cancer Centre (CNIO) | Spain, Greece | Instituto de Salud Carlos III Comité de Bioética y Bienestar Animal |
| CONsorzio Studi ITaliani sui Tumori Ereditari Alla Mammella (CONSIT TEAM) | Italy | Comitato Etico Indipendente della Fondazione IRCCS "Istituto Nazionale dei Tumori" |
| Deutsches Krebsforschungszentrum (DKFZ) | Germany | Ethik-Kommission des Klinikums der Universität |
| (DKFZ - addtional) | Columbia | Hospital Universitario de San Ignacio Comité de Investigaciones y Etica |
| (DKFZ - addtional) | Pakistan | Shaukat Khanum Memorial Cancer Hospital and Research Centre Institutional Review Board |
| HEreditary Breast and Ovarian study Netherlands (HEBON) | The Netherlands | Protocol Toetsingscommissie van het Nederlands Kanker Instituut/Antoni van Leeuwenhoek Ziekenhuis |
| Epidemiological study of BRCA1 and BRCA2 mutation carriers (EMBRACE) | UK and EIRE | Anglia & Oxford MREC |
| Fox Chase Cancer Center (FCCC) | USA | Institutional Review Board Fox Chase Cancer Center |
| German Consortium of Hereditary Breast and Ovarian Cancer (GC-HBOC) | Germany | Ethik-Kommission der Medizinischen Fakultät der Universät zu Köln |
| Georgetown University (GEORGETOWN) | USA | MedStar Research Institute - Georgetown University Oncology Institutional Review Board |
| Genetic Modifiers of cancer risk in *BRCA1*/2 mutation carriers (GEMO) | France, USA | Comité consultatif sur le traitement de I'information en matière de recherche dans le domaine de la santé |
| Hospital Clinico San Carlos (HCSC) | Spain | Comité Ético de Investigación Clínia Hospital Clínico San Carlos |
| Helsinki Breast Cancer Study (HEBCS) | Finland | Helsingin ja uudenmaan sairaanhoitopiiri (Helsinki University Central Hospital ethics committee) |
| Institut Català d'Oncologia (ICO) | Spain | Catalan Institute of Oncology Institutional Review Board |
| Iceland Landspitali - University Hospital (ILUH) | Iceland | Vísindasiđanefnd National Boethics Committee |
| Interdisciplinary Health Research International Team Breast Cancer Susceptibility (INHERIT) | Quebec -Canada | Comité d'éthique de la recherche du Centre Hospitalier Universitaire de Québec |
| Istituto Oncologico Veneto Hereditary Breast and Ovarian Cancer Study (IOVHBOCS) | Italy | Centro Oncologico Regionale Azienda Ospedale Di Padova Comitato Etico |
| Kathleen Cuningham Foundation Consortium for Research into Familial Breast Cancer (KCONFAB) | Australia | Peter MacCallum Cancer Centre Ethics Committee |
| (KCONFAB - additional) | Australia | Queensland Institute of Medical Research - Human Research Ethics Committee |
| Modifiers and Genetics in Cancer (MAGIC) | USA | University of Pennsylvania Institutional Review Board |
| Mayo Clinic (MAYO) | USA | Mayo Clinic Institutional Review Boards |
| Memorial Sloane Kettering Cancer Center (MSKCC) | USA | Memorial Sloan-Kettering Cancer Center IRB |
| (MSKCC - additional) | USA | Human Biospecimen Utilization Committee |
| Modifier Study of Quantitative Effects on Disease (MOD-SQUAD) | USA | Mayo Clinic Institutional Review Boards |
| General Hospital Vienna (MUV) | Austria | Ethikkommission der Medizinischen Universität Wien |
| National Cancer Institute (NCI) | USA | NIH Ethics Office |
| N.N. Petrov Institute of Oncology (NNPIO) | Russia | N.N. Petrov Institional Ethical Committee |
| The Ohio State University Comprehensive Cancer Centre (OSU-CCG) | USA | Cancer Institutional Review Board |
| Odense University Hospital (OUH) | Denmark | Den Videnskabsetiske Komité for Region Syddanmark |
| Swedish Breast Cancer Study (SWE-BRCA) | Sweden | Regionala Etikprövningsnämnden Stockholm |
| University of California Irvine (UCI) | USA | UC Irvine: Office of Research Administration Institutional Review Board |
| University of California San Francisco (UCSF) | USA | Committee on Human Research |
| UK and Gilda Radner Familial Ovarian Cancer Registries (UKGRFOCR) | UK | Cambridge Local Research Ethics Committee |
| (UKGRFOCR - additional) | USA | Roswell Park Cancer Institute IRB |
| University of Pennsylvania (UPENN) | USA | University of Pennsylvania Institutional Review Board |
| Women’s Cancer Research Institute (WCRI) | USA | Cedars-Sinai Institutional Review Board |

| **Supplementary Table 2**: Methods and thresholds used to define the final marker variables | | | |
| --- | --- | --- | --- |
| **Study** | **Marker** | **Source** | **Definition of positive status** |
| BCFR / OCGN | ER / PR | PRep, MRec, PRev, TReg | Not available |
| BFBOCC | ER / PR | PRep, MRec | Scoring based on cell staining percentage and dye intensity |
| CBCS | ER / PR | MRec | ≥10% stained nuclei |
| CNIO | ER / PR | MRec, TMAs | ≥10% stained nuclei or Allred score >2 |
| CONSIT-TEAM | ER / PR | PRep, MRec | ≥10% stained nuclei or Allred score >2 or H-score ≥50 or >10 fmoles/mg cytosolic protein |
| DKFZ | ER / PR | PRep, MRec | ≥5% stained nuclei or >10 fmoles/mg cytosolic protein |
| EMBRACE | ER / PR | PRep, MRec, TReg | ≥10% stained nuclei or Allred score >2 or H-score ≥50 |
| FCCC | ER / PR | PRev, TReg | Not available |
| GC-HBOC | ER / PR | MRec, PRev | ≥10% stained nuclei or Remmele score ≥1 |
| GEMO | ER / PR | PRep, MRec | ≥10% stained nuclei |
| GEORGETOWN | ER / PR | PRep, MRec | ≥10% stained nuclei |
| HCSC | ER / PR | MRec, PRev | ≥10% stained nuclei or Allred score >2 |
| HEBCS | ER / PR | MRec, PRev, TMAs | ≥10% stained nuclei |
| HEBON | ER / PR | PRep | Not available |
| ICO | ER / PR |  | Not available |
| ILUH | ER | PRep | ≥8 fmoles/mg cytosolic protein |
| ILUH | PR | PRep | ≥25 fmoles/mg cytosolic protein |
| INHERIT | ER / PR |  | Not available |
| IOVHBOCS | ER / PR | PRep, MRec | As assigned by clinicians |
| KCONFAB | ER / PR | PRep, MRec, TReg, TMAs | Not available |
| MAGIC | ER / PR |  | Not available |
| MAYO | ER / PR | MRec | Not available |
| MOD-SQUAD | ER / PR |  | Not available |
| MSKCC | ER / PR | TReg | Not available |
| MUV | ER / PR | MRec, PRev | Not available |
| NCI | ER / PR | PRep, MRec | Not available |
| NNPIO | ER / PR | MRec | Not available |
| OSU-CCG | ER / PR | PRep, MRec | Not available |
| OUH | ER / PR | TReg | ≥10% stained nuclei |
| PBCS | ER / PR | PRep, MRec | ≥1% stained nuclei |
| SWE-BRCA | ER / PR | MRec | ≥10% stained nuclei |
| UCI | ER / PR | MRec, TReg | Not available |
| UCSF | ER / PR | PRep | Not available |
| UKGRFOCR | ER / PR | PRep, MRec, TReg | Not available |
| UPENN | ER / PR | PRep, MRec | Not available |
| WCRI | ER / PR | PRep, MRec | >5% stained nuclei |
| Source abbreviations:  PRep: Pathology Reports; MRec: Medical Records; PRev: Pathology Review; TReg: Tumour Registry; TMAs: Tissue Microarray  Composite scoring methods:  Allred score 0-8 (percent staining score 0-5 + intensity of staining 0-3); H-Score 0-300 (percent staining score 0-100 * intensity of staining 0-3); Remmele score 0-12 (percent staining score 0-4 * intensity of staining 0-3). | | | |

| **Supplementary Table 3**: Per-allele Hazard Ratio estimates by progesterone receptor status for *BRCA1* mutation carriers. | | | | | | | | | | | | |
| --- | --- | --- | --- | --- | --- | --- | --- | --- | --- | --- | --- | --- |
| **SNP (allele1/allele2)** | | **Unaffected** | **Affected by subtype, N (%)** | | | **PR-** | | | **PR+** | | | **p-het** |
|  |  | **N (%)** | **PR-** | **PR+** | **Unknown** | **HR** | **95%CI** | ***P-trend*** | **HR** | **95% CI** | ***P-trend*** |  |
| *FGFR2 rs2981582 (G/A)* | |  |  |  |  |  |  |  |  |  |  |  |
|  | Per-allele | 3595 | 1014 | 275 | 2654 | 0.93 | 0.87-1.00 | 0.057 | **1.29** | **1.10-1.51** | **0.0016** | **0.00069** |
|  |  |  |  |  |  |  |  |  |  |  |  |  |
| *TOX3/TNRC9 rs3803662 (C/T)* | | |  |  |  |  |  |  |  |  |  |  |
|  | Per-allele | 3485 | 996 | 264 | 2560 | 1.07 | 0.99-1.15 | 0.091 | 1.19 | 0.99-1.42 | 0.058 | 0.31 |
|  |  |  |  |  |  |  |  |  |  |  |  |  |
| *MAP3K1* rs889312 (A/C) | |  |  |  |  |  |  |  |  |  |  |  |
|  | Per-allele | 3746 | 1042 | 280 | 2737 | 0.95 | 0.88-1.03 | 0.17 | 1.09 | 0.91-1.30 | 0.35 | 0.20 |
|  |  |  |  |  |  |  |  |  |  |  |  |  |
| *LSP1* rs3817198 (T/C) | |  |  |  |  |  |  |  |  |  |  |  |
|  | Per-allele | 3996 | 1312 | 365 | 2955 | **1.09** | **1.01-1.16** | **0.017** | 1.02 | 0.88-1.18 | 0.83 | 0.45 |
|  |  |  |  |  |  |  |  |  |  |  |  |  |
| 2q35 rs13387042 (G/A) | |  |  |  |  |  |  |  |  |  |  |  |
|  | Per-allele | 3843 | 1239 | 346 | 2887 | 0.97 | 0.91-1.04 | 0.40 | **1.16** | **1.01-1.33** | **0.039** | **0.034** |
|  |  |  |  |  |  |  |  |  |  |  |  |  |
| 8q24 rs13281615 (A/G) | |  |  |  |  |  |  |  |  |  |  |  |
|  | Per-allele | 4018 | 1269 | 352 | 2957 | 0.99 | 0.92-1.06 | 0.81 | 1.09 | 0.94-1.27 | 0.26 | 0.30 |
|  |  |  |  |  |  |  |  |  |  |  |  |  |
| SLC4A7/NEK10 rs4973768 (C/T) | | |  |  |  |  |  |  |  |  |  |  |
|  | Per-allele | 4378 | 1360 | 384 | 3006 | 1.01 | 0.95-1.07 | 0.81 | 1.12 | 0.98-1.29 | 0.10 | 0.20 |
|  |  |  |  |  |  |  |  |  |  |  |  |  |
| STXBP4/COX11 rs6504950 (G/A) | | |  |  |  |  |  |  |  |  |  |  |
|  | Per-allele | 4416 | 1398 | 396 | 3040 | 1.02 | 0.95-1.09 | 0.58 | 0.96 | 0.82-1.13 | 0.62 | 0.52 |
|  |  |  |  |  |  |  |  |  |  |  |  |  |
| 5p12 rs10941679 (A/G) | | |  |  |  |  |  |  |  |  |  |  |
|  | Per-allele | 3963 | 1313 | 373 | 2887 | 0.95 | 0.87-1.02 | 0.16 | 0.94 | 0.80-1.11 | 0.48 | 0.97 |
|  |  |  |  |  |  |  |  |  |  |  |  |  |
| 6q25.1 - rs2046210 (C/T) | |  |  |  |  |  |  |  |  |  |  |  |
|  | Per-allele | 4352 | 1334 | 378 | 2934 | **1.19** | **1.11-1.27** | **3.7×10^-7^** | **1.14** | **0.99-1.30** | **0.064** | 0.61 |
|  |  |  |  |  |  |  |  |  |  |  |  |  |
| 1p11.2 - rs11249433 (T/C) | |  |  |  |  |  |  |  |  |  |  |  |
|  | Per-allele | 4376 | 1353 | 377 | 493 | 1.01 | 0.95-1.08 | 0.75 | **0.86** | **0.74-0.99** | **0.034** | 0.053 |
|  |  |  |  |  |  |  |  |  |  |  |  |  |
| *RAD51L1* - rs999737 / rs10483813 (C/T, T/A) | | | |  |  |  |  |  |  |  |  |  |
|  | Per-allele | 3799 | 1052 | 270 | 2683 | 1.02 | 0.94-1.11 | 0.64 | **0.76** | **0.61-0.95** | **0.017** | **0.027** |
| p-het: Heterogeneity p-value; PR-: Progesterone receptor negative; PR+: Progesterone receptor positive | | | | | | | | | | | | |

| **Supplementary Table 4**: Per-allele Hazard Ratio estimates by progesterone receptor status for *BRCA2* mutation carriers. | | | | | | | | | | | | |
| --- | --- | --- | --- | --- | --- | --- | --- | --- | --- | --- | --- | --- |
|  | **Genotype** | **Unaffected,** | **Affected by subtype, N (%)** | | | **PR-** | | | **PR+** | | | **p-diff** |
|  |  | **N (%)** | **PR-** | **PR+** | **Unknown** | **HR** | **95%CI** | ***P-trend*** | **HR** | **95% CI** | ***P-trend*** |  |
| *FGFR2 rs2981582 (G/A)* | |  |  |  |  |  |  |  |  |  |  |  |
|  | Per-allele | 2102 | 364 | 592 | 1680 | **1.21** | **1.05-1.39** | **0.0079** | **1.35** | **1.22-1.50** | **1.6×10^-8^** | 0.24 |
|  |  |  |  |  |  |  |  |  |  |  |  |  |
| *TOX3/TNRC9 rs3803662 (C/T)* | | |  |  |  |  |  |  |  |  |  |  |
|  | Per-allele | 2037 | 353 | 568 | 1605 | **1.20** | **1.03-1.40** | **0.019** | **1.24** | **1.20-1.39** | **0.00032** | 0.79 |
|  |  |  |  |  |  |  |  |  |  |  |  |  |
| *MAP3K1* rs889312 (A/C) | |  |  |  |  |  |  |  |  |  |  |  |
|  | Per-allele | 2165 | 370 | 603 | 1711 | 0.99 | 0.84-1.16 | 0.89 | **1.20** | **1.07-1.34** | **0.0017** | 0.072 |
|  |  |  |  |  |  |  |  |  |  |  |  |  |
| *LSP1* rs3817198 (T/C) | |  |  |  |  |  |  |  |  |  |  |  |
|  | Per-allele | 2332 | 445 | 728 | 1849 | **1.19** | **1.04-1.36** | **0.012** | **1.11** | **1.00-1.23** | **0.05** | 0.43 |
|  |  |  |  |  |  |  |  |  |  |  |  |  |
| 2q35 rs13387042 (G/A) | |  |  |  |  |  |  |  |  |  |  |  |
|  | Per-allele | 2259 | 434 | 694 | 1822 | 0.92 | 0.81-1.04 | 0.19 | **1.14** | **1.03-1.25** | **0.0081** | **0.0086** |
|  |  |  |  |  |  |  |  |  |  |  |  |  |
| 8q24 rs13281615 (A/G) | |  |  |  |  |  |  |  |  |  |  |  |
|  | Per-allele | 2332 | 438 | 710 | 1841 | 1.06 | 0.93-1.21 | 0.39 | 1.05 | 0.95-1.16 | 0.32 | 0.94 |
|  |  |  |  |  |  |  |  |  |  |  |  |  |
| SLC4A7/NEK10 rs4973768 (C/T) | | |  |  |  |  |  |  |  |  |  |  |
|  | Per-allele | 2528 | 470 | 788 | 1886 | 1.07 | 0.94-1.21 | 0.29 | **1.12** | **1.02-1.23** | **0.016** | 0.57 |
|  |  |  |  |  |  |  |  |  |  |  |  |  |
| STXBP4/COX11 rs6504950 (G/A) | | |  |  |  |  |  |  |  |  |  |  |
|  | Per-allele | 2555 | 478 | 804 | 1891 | 1.08 | 0.94-1.22 | 0.27 | 1.06 | 0.96-1.17 | 0.28 | 0.85 |
|  |  |  |  |  |  |  |  |  |  |  |  |  |
| 5p12 rs10941679 (A/G) | | |  |  |  |  |  |  |  |  |  |  |
|  | Per-allele | 2342 | 454 | 773 | 1800 | 0.94 | 0.81-1.09 | 0.40 | **1.15** | **1.03-1.27** | **0.0095** | **0.028** |
|  |  |  |  |  |  |  |  |  |  |  |  |  |
| 6q25.1 - rs2046210 (C/T) | |  |  |  |  |  |  |  |  |  |  |  |
|  | Per-allele | 2474 | 446 | 786 | 1866 | 1.13 | 0.98-1.29 | 0.08 | 0.95 | 0.86-1.04 | 0.26 | 0.053 |
|  |  |  |  |  |  |  |  |  |  |  |  |  |
| 1p11.2 - rs11249433 (T/C) | |  |  |  |  |  |  |  |  |  |  |  |
|  | Per-allele | 2492 | 485 | 776 | 1908 | **1.16** | **1.01-1.32** | **0.03** | 1.06 | 0.96-1.16 | 0.23 | 0.32 |
|  |  |  |  |  |  |  |  |  |  |  |  |  |
| *RAD51L1* - rs999737 / rs10483813 (C/T, T/A) | | | |  |  |  |  |  |  |  |  |  |
|  | Per-allele | 2298 | 396 | 671 | 1790 | 1.01 | 0.86-1.18 | 0.92 | 0.91 | 0.81-1.02 | 0.10 | 0.33 |
| p-het: Heterogeneity p-value; PR-: Progesterone receptor negative; PR+: Progesterone receptor positive | | | | | | | | | | | | |
